# Supplementary material for: Multiplexed ddPCR-amplicon sequencing reveals isolated Plasmodium falciparum populations amenable to local elimination in Zanzibar, Tanzania
Source: Nat Commun. 2023 Jun 22;14:3699. doi: 10.1038/s41467-023-39417-1 (PMC10287761; doi:10.1038/s41467-023-39417-1)
Supplement: Supplementary file 1 — Supplemetary Information [file 41467_2023_39417_MOESM1_ESM.pdf]

SUPPLEMENTARY INFORMATION

Supplementary information for the paper: Holzschuh et al. *Multiplexed ddPCR-amplicon sequencing reveals isolated Plasmodium falciparum populations amenable to local elimination in Zanzibar, Tanzania*

TABLE OF CONTENTS

1. Supplementary Table 1	Page 2
2. Supplementary Table 2	Page 3
3. Supplementary Table 3	Page 4
4. Supplementary Table 4	Page 5
5. Supplementary Table 5	Page 6
6. Supplementary Table 6	Page 7
7. Supplementary Table 7	Page 8
8. Supplementary Table 8	Page 9
9. Supplementary Table 9	Page 10
10. Supplementary Table 10	Page 11
11. Supplementary Table 11	Page 12
12. Supplementary Table 12	Page 13
13. Supplementary Table 13	Page 14
14. Supplementary Table 14	Page 15
15. Supplementary Table 15	Page 18
16. Supplementary Table 16	Page 18
17. Supplementary Table 17	Page 19
18. Supplementary Table 18	Page 20
19. Supplementary Figure 1	Page 25
20. Supplementary Figure 2	Page 26
21. Supplementary Figure 3	Page 27
22. Supplementary Figure 4	Page 28
23. Supplementary Figure 5	Page 29
24. Supplementary Figure 6	Page 30
25. Supplementary Figure 7	Page 31
26. Supplementary Figure 8	Page 32
27. Supplementary Figure 9	Page 33
28. Supplementary Figure 10	Page 34
29. Supplementary Figure 11	Page 35
30. Supplementary Figure 12	Page 36
31. Supplementary Figure 13	Page 37
32. Supplementary Figure 14	Page 38
33. Supplementary Figure 15	Page 39
34. Supplementary Figure 16	Page 40
35. Supplementary Figure 17	Page 41
36. Supplementary Figure 18	Page 42
37. Supplementary Figure 19	Page 43
References	Page 44

SUPPLEMENTARY TABLES

Supplementary Table 1. Number of reads for whole blood and dried blood spot (DBS) control samples.

Sample	Parasite density (per µL)	*n*	Median reads (range) [a]	Median reads per locus (range) [a]
**Whole blood**		20	491,561 (77,531 – 979,755)	
	10,000	5		6,830 (266 – 61,834)
	1,000	5		5,387 (183 – 86,854)
	100	5		5,237 (132 – 63,154)
	10	5		2,225 (62 – 32,765)
**Dried blood spots**		13	251,168 (33,307 – 880,596)	
	1,000	3		11,244 (591 – 103,714)
	100	5		2,408 (83 – 36,666)
	10	5		472 (0 – 16,517)

[a] after trimming of low-quality reads.

Supplementary Table 2. Detection of minority clone in mixed strain infections from cultured *P. falciparum* parasites at different ratios and parasite densities.

Strain mixtures	Abundance of minority clone	Parasite density (per µL)	Expected number of minority clones per µL to be detected	% of heterozygotes detected in minority clone (n/N) with cut-off [a]				% of heterozygotes detected in minority clone (n/N) without cut-off [b]			
				Rep 1	Rep 2	Rep 3	Rep 4	Rep 1	Rep 2	Rep 3	Rep 4
**3D7:FCB**	40%	1,000	400	100.0% (27/27)	100.0% (27/27)	-	-	100.0% (27/27)	100.0% (27/27)	-	-
	40%	100	40	100.0% (27/27)	100.0% (27/27)	-	-	100.0% (27/27)	100.0% (27/27)	-	-
	40%	10	4	63.0% (17/27)	92.6% (25/27)	-	-	63.0% (17/27)	92.6% (25/27)	-	-
	20%	1,000	200	70.4% (19/27)	70.4% (19/27)	-	-	74.1% (20/27)	77.8% (21/27)	-	-
	20%	100	20	66.7% (18/27)	77.8% (21/27)	-	-	77.8% (21/27)	77.8% (21/27)	-	-
	20%	10	2	48.1% (13/27)	66.7% (18/27)	-	-	48.1% (13/27)	70.4% (19/27)	-	-
**3D7:HB3**	2%	1,000	20	52.4% (11/21)	61.9% (13/21)	66.7% (14/21)	71.4% (15/21)	71.4% (15/21)	71.4% (15/21)	71.4% (15/21)	71.4% (15/21)
	1%	1,000	10	28.6% (6/21)	33.3% (7/21)	33.3% (7/21)	42.9% (9/21)	47.6% (10/21)	61.9% (13/21)	66.7% (14/21)	47.6% (10/21)

[a] "With cut-off" meaning a within-host haplotype frequency of ≥1%.
[b] "No cut-off" meaning a within-host haplotype frequency of ≥0.1%.

Supplementary Table 3. Comparison between ddPCR and conventional PCR. Table shows %coverage of markers, fold-difference between the highest number of reads and lowest number of reads per sample, and %reads lost after trimming between the two methods on range of different parasite densities (500, 50, and 5 parasites/µL; *P. falciparum* culture line 3D7 was used).

PCR method	Parasite density (per µL)	%coverage of markers (out of 35 total)		Fold-difference between the highest number of reads and lowest number of reads per sample (range)		Mean number of reads per sample (±SD)		%reads lost per sample after trimming of low-quality reads.	
		Rep1	Rep2	Rep 1	Rep 2	Rep 1	Rep 2	Rep 1	Rep 2
**ddPCR**	500	100% (35/35)	100% (35/35)	45X (314 – 14,233)	198X (96 – 19’053)	3,991 (±4,263)	3,130 (±4,470)	3.8% (6,044/158,429)	4.3% (5,333/124,390)
	50	100% (35/35)	100% (35/35)	23X (465 – 11,118)	98X (102 – 10,004)	3,571 (±2,600)	3,283 (±2,647)	4.5% (6,463/144,344)	4.2% (5,762/135,725)
	5	100% (35/35)	100% (35/35)	182X (31 – 5,661)	220X (32 – 7,048)	1,611 (±1,521)	1,622 (±1,728)	4.1% (2,331/56,757)	5.1% (3,011/59,166)
**Conventional PCR**	500	100% (35/35)	100% (35/35)	863X (24 – 20,733)	1,035X (139 – 143,911)	3,590 (±4,832)	24,897 (±34,050)	4.8% (7,006/144,481)	5.7% (57,602/1,013,385)
	50	100% (35/35)	100% (35/35)	456X (243 – 110,876)	485X (157 – 76,106)	27,842 (±25,304)	18,360 (±17,638)	6.1% (70,469/1,146,922)	6.2% (47,222/760,371)
	5	100% (35/35)	100% (35/35)	429X (307 – 131,617)	511X (150 – 76,654)	34,086 (±29,502)	18,256 (±17,073)	10.2% (150,907/1,473,662)	11.0% (87,448/798,093)

Supplementary Table 4. Number of samples included in further analyses (290/518) by districts. No significant difference in proportion of samples included by districts.

Population	Samples included	*P value* [a]	*Chi-square statistic*
**Districts**		0.6395	2.5285
**Micheweni**	59.3% (57/96)		
**Chake Chake**	50.0% (43/86)		
**Mkoani**	58.8% (20/34)		
**Magharibi**	57.9% (121/209)		
**Kusini**	52.7% (49/93)		

[a] *P*-value were two-tailed and computed using the chi-squared test for comparing the proportion of samples by district.

Supplementary Table 5. Breakdown of samples successfully sequenced (*n*=290) from the same vs. different households by district and year. No clear differences between Unguja and Pemba were observed.

District	n	Same households (%)	Different households (%)
Pemba			
Micheweni 2017	45	24 (53.3%)	21 (46.7%)
Micheweni 2018	12	4 (33.3%)	8 (66.7%)
Chake Chake 2017	27	9 (33.3%)	18 (66.7%)
Chake Chake 2018	16	4 (25.0%)	12 (75.0%)
Mkoani 2017	11	4 (36.4%)	7 (63.6%)
Mkoani 2018	9	5 (55.6%)	4 (44.4%)
Unguja			
Magharibi 2017	44	24 (54.5%)	20 (45.5%)
Magharibi 2018	77	51 (66.2%)	26 (33.8%)
Kusini 2017	27	11 (40.7%)	16 (59.3%)
Kusini 2018	22	12 (54.5%)	10 (45.5%)

Supplementary Table 6. Estimates of multilocus linkage disequilibrium (mLD) for *P. falciparum* populations in Zanzibar. *P* values were calculated by applying a Monte Carlo test with 100,000 re-sampling steps, as described elsewhere [1]. I_A^S = standardized index of association.

Population	All infections		Monoclonal infections	
	n	**I_A^S (*P* value)**	***n***	**I_A^S (*P* value)**
**Pemba Island**	51	0.0691 (<1e-5)	14	0.0847 (<1e-5)
**Micheweni**	25	0.1485 (<1e-5)	7	0.2682 (<1e-5)
**Chake Chake**	19	0.1402 (<1e-5)	6	0.1264 (<1e-5)
**Mkoani**	7	0.0056 (0.355)	1	NA
**Unguja Island**	73	0.0077 (<1e-5)	14	0.0376 (<1e-5)
**Magharibi**	57	0.0054 (0.02)	9	-0.0022 (0.606)
**Kusini**	16	0.0582 (<1e-5)	5	0.3437 (<1e-5)
**All**	124	0.0168 (<1e-5)	28	0.0356 (<1e-5)

Supplementary Table 7. Mean IBD and proportion of related infection pairs in Zanzibar, stratified by island, district, shehia, RACD cluster, and year. *P* values of each comparison were calculated by one-sided permutation test (100,000 permutations).

Comparisons	Mean IBD	Fold change	*P value*	Proportion related infections	Fold change	*P value*
**Island**		2.33	< 1e-5		2	< 1e-5
**Pemba Island**	0.07			0.08		
**Unguja Island**	0.03			0.04		
**District**		2	< 1e-5		3	< 1e-5
**Within**	0.06			0.09		
**Between**	0.03			0.03		
Shehia		2.25	< 1e-5		1.75	< 1e-5
**Within**	0.09			0.07		-
**Between**	0.04			0.04		-
**RACD cluster**		3.25	< 1e-5		1.13	< 1e-5
**Within**	0.13			0.043		
**Between**	0.04			0.038		
**Year**		1.33	< 1e-5		1.33	< 1e-5
**Within**	0.04			0.04		
**Between**	0.03			0.03		
		1.66	< 1e-5		1.25	0.0003
**2017**	0.05			0.05		
**2018**	0.03			0.04		
**Travel history**		1.33	0.003		1.66	0.0026
**Non-travelers**	0.04			0.05		
**Travelers**	0.03			0.03		

Supplementary Table 8. Mean IBD and proportion of related infection pairs between Zanzibar and mainland Tanzania/Kenya, stratified by island, district and travel/no travel. *P* values of each comparison between travel vs. non-travel were calculated by one-sided permutation test (100,000 permutations).

Pairs	*n* pairs	Mean IBD	*P value*	Proportion related infections	*P value*
**Zanzibar – Tanzania/Kenya**	33,350	0.023	-	0.029	-
**Zanzibar – Tanzania**	23,200	0.024	-	0.031	-
**Zanzibar – Kenya**	10,150	0.022	-	0.024	-
**Unguja Island – Tanzania/Kenya**	19,550	0.025	-	0.034	-
**Travel**	7,245	0.025	0.5804	0.031	0.9366
**No travel**	12,305	0.025		0.035	
**Magharibi**	13,915	0.025	-	0.034	-
**Travel**	4,485	0.026	0.2809	0.032	0.7717
**No travel**	9,430	0.025		0.035	
**Kusini**	5,635	0.024	-	0.032	-
**Travel**	2,760	0.023	0.8187	0.028	0.9224
**No travel**	2,875	0.025		0.037	
**Pemba Island – Tanzania/Kenya**	13,800	0.021	-	0.023	-
**Travel**	1,725	0.026	**0.0041**	0.026	0.3043
**No travel**	12,075	0.020		0.023	
**Micheweni**	6,555	0.022	-	0.022	-
**Travel**	575	0.024	0.2227	0.017	0.8068
**No travel**	5,980	0.022		0.023	
**Chake Chake**	4,945	0.018	-	0.020	-
**Travel**	805	0.024	**0.0185**	0.030	0.0561
**No travel**	4,140	0.017		0.018	
**Mkoani**	2,300	0.026	-	0.033	-
**Travel**	345	0.037	**0.0062**	0.029	0.6917
**No Travel**	1,955	0.024		0.034	

Supplementary Table 9. Mean pairwise IBD and proportion of related infection pairs between samples from Zanzibar and several African and Asian countries.

Pairs with Zanzibar		*n* pairs	Mean IBD	Proportion related infections	Proportion related infections with IBD ≥0.25
**Asia**					
	**Bangladesh**	2,900	0.007	0.004	0.001
	**Myanmar**	2,900	0.005	0.003	0.0003
	**Thailand**	2,900	0.004	0.004	0
	**Laos**	2,900	0.005	0.004	0
	**Cambodia**	2,610	0.008	0.007	0.0008
	**Vietnam**	2,610	0.005	0.005	0
**West Africa**					
	**Gambia**	2,900	0.018	0.028	0.0086
	**Guinea**	2,610	0.019	0.027	0.0031
	**Mali**	2,900	0.018	0.023	0.0041
	**Ghana**	2,900	0.017	0.022	0.0031
	**Nigeria**	2,900	0.017	0.022	0.0031
**Central Africa**					
	**DRC**	2,900	0.026	0.032	0.0052
**East Africa**					
	**Kenya**	10,150	0.025	0.031	0.0082
	**Malawi**	2,900	0.029	0.036	0.0069
	**Tanzania**	23,200	0.028	0.040	0.0088
	**Zanzibar**	41,905	0.045	0.052	0.0293

Supplementary Table 10. Mean pairwise IBD and proportion of related infection within sites from several African and southeast Asian countries. *P* values of each within relatedness comparisons were calculated by one-sided permutation test (100,000 permutations).

Pairs within sites	Mean IBD	*P value*	Proportion significantly related infections	*P value*
Southeast Asia				
Bangladesh	0.1762	0.00002	0.7200	< 1e-5
Myanmar	0.2377	< 1e-5	0.8600	< 1e-5
Thailand	0.2913	< 1e-5	0.8200	< 1e-5
Laos	0.2194	< 1e-5	0.7200	< 1e-5
Cambodia	0.1637	0.00006	0.4691	< 1e-5
Vietnam	0.2948	< 1e-5	0.5432	< 1e-5
West Africa				
Gambia	0.1452	0.00011	0.2600	0.00011
Guinea	0.0425	0.13757	0.0494	0.45338
Mali	0.0571	0.02930	0.1600	0.00287
Ghana	0.0914	0.00249	0.2000	0.00074
Nigeria	0.0464	0.08012	0.1400	0.00759
Central Africa				
DRC	0.0302	0.38064	0.0400	0.57333
East Africa				
Kenya	0.0283	0.50834	0.0571	0.07680
Malawi	0.0379	0.18783	0.0400	0.57536
Tanzania	0.0262	0.89018	0.0434	0.43101

Supplementary Table 11. Frequency of mutations from drug resistance alleles in the population stratified by district. All *P* values were two-tailed and computed using the Pearson's Chi-squared test with simulated *P* value (based on 2000 replicates) for comparing the proportion of mutant alleles.

Gene	Mutation	Frequency% (*n/N*)					*P* value
		Micheweni	Chake Chake	Mkoani	Magharibi	Kusini	
pfdhfr	C50R	0% (0/38)	0% (0/37)	0% (0/18)	0% (0/102)	0% (0/36)	NA
	**N51I**	**100% (38/38)**	**97.3% (36/37)**	**83.3% (15/18)**	**96.1% (98/102)**	**97.2% (35/36)**	**0.048**
	C59R	89.5% (34/38)	89.2% (33/37)	72.2% (13/18)	86.3% (88/102)	97.2% (35/36)	0.1124
	S108N	100% (38/38)	100% (37/37)	100% (18/18)	100% (102/102)	100% (36/36)	NA
	S108T	0% (0/38)	0% (0/37)	0% (0/18)	0% (0/102)	0% (0/36)	NA
	I164L	0% (0/47)	0% (0/38)	0% (0/19)	0% (0/110)	0% (0/41)	NA
pfdhps	**K540E**	**95.3% (41/43)**	**47.2% (17/36)**	**68.8% (11/16)**	**92.6% (100/108)**	**83.3% (30/36)**	**0.0005**
	A581G	0% (0/43)	0% (0/36)	0% (0/16)	0% (0/108)	0% (0/36)	NA
	A613T	0% (0/43)	0% (0/36)	0% (0/16)	0% (0/108)	0% (0/36)	NA
	A613S	0% (0/43)	0% (0/36)	0% (0/16)	0% (0/108)	0% (0/36)	NA
pfmdr1	N86Y	2.4% (1/42)	5.7% (2/35)	0% (0/19)	2.8% (3/106)	7.3% (3/41)	0.5967
	Y184F	68.5% (37/54)	69.0% (29/42)	63.2% (12/19)	73.9% (85/118)	48.9% (23/47)	0.084
pfmdr2	I492V	37.3% (19/51)	27.0% (10/37)	44.4% (8/18)	48.7% (56/115)	47.6% (20/42)	0.1759
	T484I	0% (0/51)	0% (0/37)	0% (0/18)	0% (0/115)	0% (0/42)	NA
pfk13	I543T	0% (0/52)	0% (0/40)	0% (0/17)	0% (0/114)	0% (0/42)	NA
	R539T	0% (0/52)	0% (0/40)	0% (0/17)	0% (0/114)	0% (0/42)	NA
	G538V	0% (0/52)	0% (0/40)	0% (0/17)	0% (0/114)	0% (0/42)	NA
	N537I	0% (0/52)	0% (0/40)	0% (0/17)	0% (0/114)	0% (0/42)	NA
	P527H	0% (0/52)	0% (0/40)	0% (0/17)	0% (0/114)	0% (0/42)	NA
	Y493H	0% (0/52)	0% (0/40)	0% (0/17)	0% (0/114)	0% (0/42)	NA
	A481V	0% (0/52)	0% (0/40)	0% (0/17)	0% (0/114)	0% (0/42)	NA

Supplementary Table 12. Overview and genomic location of the 35 loci included in the panel.

name	seqnames	start	end	width	type	geneID	description
t01	Pf3D7_01_v3	145417	145655	239	Microhaplotype	PF3D7_0103300	conserved protein, unknown function
cpmp_22	Pf3D7_01_v3	180130	180370	241	Microhaplotype	PF3D7_0104100	conserved Plasmodium membrane protein, unknown function
t04	Pf3D7_01_v3	495942	496171	230	Microhaplotype	PF3D7_0113100	surface-associated interspersed protein 1.1 (SURFIN 1.1)
t08	Pf3D7_01_v3	534183	534397	215	Microhaplotype	PF3D7_0113800	DBL containing protein, unknown function
t11	Pf3D7_02_v3	278145	278369	225	Microhaplotype	PF3D7_0207000	merozoite surface protein 4
csp_27	Pf3D7_03_v3	221468	221655	188	Microhaplotype	PF3D7_0304600	circumsporozoite (CS) protein
t17	Pf3D7_03_v3	618368	618613	246	Microhaplotype	PF3D7_0315200	circumsporozoite- and TRAP-related protein
t25	Pf3D7_04_v3	748201	748469	269	Drug Resistance	PF3D7_0417200	bifunctional dihydrofolate reductase-thymidylate synthase
t26	Pf3D7_04_v3	748501	748725	225	Drug Resistance	PF3D7_0417200	bifunctional dihydrofolate reductase-thymidylate synthase
t28	Pf3D7_04_v3	1037603	1037868	266	Microhaplotype	PF3D7_0422500	pre-mRNA-splicing helicase BRR2, putative
t30	Pf3D7_04_v3	1102363	1102606	244	Microhaplotype	PF3D7_0424400	surface-associated interspersed protein 4.2 (SURFIN 4.2)
t31	Pf3D7_04_v3	1113423	1113632	210	Microhaplotype	PF3D7_0424600	Plasmodium exported protein (PHISTb), unknown function
t33	Pf3D7_05_v3	329352	329578	227	Microhaplotype	PF3D7_0508000	6-cysteine protein
t34	Pf3D7_05_v3	958028	958254	227	Drug Resistance	PF3D7_0523000	multidrug resistance protein 1
t35	Pf3D7_05_v3	958363	958539	177	Drug Resistance	PF3D7_0523000	multidrug resistance protein 1
t45	Pf3D7_07_v3	1358667	1358941	275	Microhaplotype	PF3D7_0731500	erythrocyte binding antigen-175
t47	Pf3D7_08_v3	336439	336674	236	Microhaplotype	PF3D7_0806200	Dpy-19-like C-mannosyltransferase, putative
t49	Pf3D7_08_v3	549963	550251	289	Drug Resistance	PF3D7_0810800	hydroxymethyldihydropterin pyrophosphokinase-dihydropteroate synthase
t53	Pf3D7_08_v3	1362864	1363120	257	Microhaplotype	PF3D7_0831600	cytoadherence linked asexual protein 8
t54	Pf3D7_09_v3	516905	517125	221	Microhaplotype	PF3D7_0911300	cysteine repeat modular protein 1
t60	Pf3D7_10_v3	377063	377237	175	Microhaplotype	PF3D7_1009200	ribonuclease, putative
t62	Pf3D7_10_v3	1386673	1386902	230	Microhaplotype	PF3D7_1035000	U2 snRNA/tRNA pseudouridine synthase, putative
t66	Pf3D7_11_v3	1009832	1010071	240	Microhaplotype	PF3D7_1125800	kelch domain-containing protein, putative
t67	Pf3D7_11_v3	1018921	1019113	193	Microhaplotype	PF3D7_1126100	autophagy-related protein 7, putative
ama1_D2_18	Pf3D7_11_v3	1294271	1294520	250	Microhaplotype	PF3D7_1133400	apical membrane antigen 1
t72	Pf3D7_12_v3	63136	63313	178	Microhaplotype	PF3D7_1200700	acyl-CoA synthetase
t73	Pf3D7_12_v3	659859	660037	179	Microhaplotype	PF3D7_1216600	cell traversal protein for ookinetes and sporozoites
t74	Pf3D7_12_v3	684060	684289	230	Microhaplotype	PF3D7_1217300	GTP-binding protein EngA
msp7_1	Pf3D7_13_v3	1419369	1419544	176	Microhaplotype	PF3D7_1335100	merozoite surface protein 7
t82	Pf3D7_13_v3	1725338	1725592	255	Drug Resistance	PF3D7_1343700	kelch protein K13
t85	Pf3D7_13_v3	2124603	2124876	274	Microhaplotype	PF3D7_1353100	Plasmodium exported protein, unknown function
t96	Pf3D7_14_v3	1956097	1956312	216	Drug Resistance	PF3D7_1447900	multidrug resistance protein 2
t98	Pf3D7_14_v3	2524932	2525119	188	Microhaplotype	PF3D7_1462300	GTP-binding protein, putative
cpp_30	Pf3D7_14_v3	3121036	3121267	232	Microhaplotype	PF3D7_1475800	conserved Plasmodium protein, unknown function
t99	Pf3D7_14_v3	3124615	3124870	256	Microhaplotype	PF3D7_1475900	KELT protein

Supplementary Table 13. Expected heterozygosity for each marker calculated from the global Pf7 dataset from MalariaGEN[2] for all samples (global), samples from Tanzania only, and samples from East Africa. The total number of haplotypes (numHaplotypes) and number of SNPs (numSNP) for each marker is shown as well (for the global population only).

Marker	Global_He	Tanzania_He	EastAfrica_He	numHaplotypes	numSNP
ama1_D2_18	0.95404221	0.95338843	0.95237179	151	49
cpmp_22	0.95221289	0.971293008	0.97354227	460	67
cpp_30	0.56396197	0.294494772	0.29273988	44	30
csp_27	0.90170864	0.947587877	0.94342053	140	52
msp7_1	0.79696477	0.748051845	0.73500779	49	30
t01	0.60027898	0.598638767	0.62350256	31	51
t04	0.91834553	0.928288127	0.93352885	246	88
t08	0.68606452	0.661257413	0.68040871	64	62
t11	0.8628728	0.88806303	0.87533234	102	60
t17	0.66139123	0.49694521	0.50567374	16	23
t25	0.34613212	0.056702013	0.16708933	12	33
t26	0.33914083	0.003144646	0.00233918	5	20
t28	0.5036624	0.44796908	0.45320332	42	66
t30	0.84206428	0.810350407	0.80209166	86	58
t31	0.00259993	0	0.00350398	11	25
t33	0.73047929	0.790499959	0.78996421	58	51
t34	0.19907883	0.216220323	0.40055278	13	24
t35	0.49970819	0.452977778	0.43036915	5	18
t45	0.69659255	0.640528996	0.66674702	21	23
t47	0.40360596	0.549789044	0.56896107	25	50
t49	0.66044431	0.531174108	0.43448305	29	33
t53	0.79506627	0.762854938	0.74929754	32	58
t54	0.70083374	0.583693827	0.54891322	34	44
t60	0.0048017	0.006206328	0.00813947	16	24
t62	0.65087151	0.456097254	0.4770043	51	62
t66	0.65732621	0.265969935	0.23051384	23	45
t67	0.65150099	0.440036693	0.49471886	23	45
t72	0.50226718	0.498812667	0.50590152	9	24
t73	0.92710698	0.944749321	0.94086502	196	49
t74	0.78564304	0.468909046	0.50204963	47	42
t82	0.05077982	0.012693035	0.01064023	36	74
t85	0.73336521	0.253571244	0.21929866	23	50
t96	0.6463372	0.424855821	0.37745737	24	57
t98	0.55280543	0.47973514	0.48016402	37	46
t99	0.83759193	0.87968214	0.86571499	87	55

Supplementary Table 14. Primer sequences for the 35-plex panel. Final primer pools are generated by combining 10µL of each primer stock (total 700 µL) with 300 µL NF ddH2O. This is then used to prepare the PCR reaction.

Primer	Direction	Sequence (5' - 3')	Source	Stock concentration (µM)	Concentration in final pool (µM)	Final concentration in PCR reaction (µM)
ama1_D2_18	Forward	GTGACCTATGAACTCAGGAGTCGAACTCAATATAGACTTCCATCAGG	This study	1000	10	0.227
cpmp_22	Forward	GTGACCTATGAACTCAGGAGTCGGAAGCTATAGGTATCAGATCC	This study	1000	10	0.227
cpp_30	Forward	GTGACCTATGAACTCAGGAGTCAACACAATCTTCCTTAGCCAATTC	This study	1000	10	0.227
csp_27	Forward	GTGACCTATGAACTCAGGAGTCGACCCAAACCGAAATGTAGATG	This study	1000	10	0.227
msp7_1	Forward	GTGACCTATGAACTCAGGAGTCGCACAAGGAGGAGAATCGAC	This study	1000	10	0.227
t01	Forward	GTGACCTATGAACTCAGGAGTCTTCGATATGTTTAAATATATGATTCTCG	Tessema et al., 2020	1000	10	0.227
t11	Forward	GTGACCTATGAACTCAGGAGTCCTTACCATTCGCGCTTTCTTG	Tessema et al., 2020	1000	10	0.227
t17	Forward	GTGACCTATGAACTCAGGAGTCGACACTACAGACAAAAATAAATGATC	Tessema et al., 2020	1000	10	0.227
t25	Forward	GTGACCTATGAACTCAGGAGTCCTAGGAAATAAAGGAGTATTACCATG	Tessema et al., 2020	1000	10	0.227
t26	Forward	GTGACCTATGAACTCAGGAGTCTGTTTATATCATTAACAAAGTTGAAGATC	Tessema et al., 2020	1000	10	0.227
t28	Forward	GTGACCTATGAACTCAGGAGTCTTGAAGTACAATATGAAATCGATCTTG	Tessema et al., 2020	1000	10	0.227
t30	Forward	GTGACCTATGAACTCAGGAGTCTAGAGGTGTTGATGTTAATATGGAG	Tessema et al., 2020	1000	10	0.227
t31	Forward	GTGACCTATGAACTCAGGAGTCTCTGCATGCAGTAATGAATCTATTG	Tessema et al., 2020	1000	10	0.227
t33	Forward	GTGACCTATGAACTCAGGAGTCGTTAAAACAACAGGAGGAACACTAA	Tessema et al., 2020	1000	10	0.227
t34	Forward	GTGACCTATGAACTCAGGAGTCAAATGTTTACCTGCACAACATAGAAA	Tessema et al., 2020	1000	10	0.227
t35	Forward	GTGACCTATGAACTCAGGAGTCGAACAAGTGAGTTCAGGAATTGG	Tessema et al., 2020	1000	10	0.227
t04	Forward	GTGACCTATGAACTCAGGAGTCCACCAAAATATTATATACCACAAGAC	Tessema et al., 2020	1000	10	0.227
t45	Forward	GTGACCTATGAACTCAGGAGTCAAGGATCATTTCATTGAAGCCTCT	Tessema et al., 2020	1000	10	0.227
t47	Forward	GTGACCTATGAACTCAGGAGTCATACATGAATATGATTAACAACGAACC	Tessema et al., 2020	1000	10	0.227
t49	Forward	GTGACCTATGAACTCAGGAGTCAAAAGAGGAAATCCACATACAATGG	Tessema et al., 2020	1000	10	0.227
t53	Forward	GTGACCTATGAACTCAGGAGTCCGACTTCTTTAAGAGAACAAACAG	Tessema et al., 2020	1000	10	0.227
t54	Forward	GTGACCTATGAACTCAGGAGTCTGTGGGCGCAAAAACTATAAATGA	Tessema et al., 2020	1000	10	0.227

t60	Forward	GTGACCTATGAACTCAGGAGTCCAAATAGCATTCGATAAATTTGAAAATTTC	Tessema et al., 2020	1000	10	0.227
t62	Forward	GTGACCTATGAACTCAGGAGTCCTACAGTTATATAGGACGATTTTGG	Tessema et al., 2020	1000	10	0.227
t66	Forward	GTGACCTATGAACTCAGGAGTCCTGTGAGCATATCTTGGTTCAG	Tessema et al., 2020	1000	10	0.227
t67	Forward	GTGACCTATGAACTCAGGAGTCTACTACTACTTATGTTACTTATACCAC	Tessema et al., 2020	1000	10	0.227
t72	Forward	GTGACCTATGAACTCAGGAGTCATTCTATTAAAAACTTTGGGTACTCC	Tessema et al., 2020	1000	10	0.227
t73	Forward	GTGACCTATGAACTCAGGAGTCCTGGTACTATTATACCATATGTTGC	Tessema et al., 2020	1000	10	0.227
t74	Forward	GTGACCTATGAACTCAGGAGTCCTACCATTTTTAATCGACTAACTCG	Tessema et al., 2020	1000	10	0.227
t08	Forward	GTGACCTATGAACTCAGGAGTCCAGATGTGATAAATATATGTGACATTTG	Tessema et al., 2020	1000	10	0.227
t82	Forward	GTGACCTATGAACTCAGGAGTCGTATAATAGAAGAGCCATCATATCC	Tessema et al., 2020	1000	10	0.227
t85	Forward	GTGACCTATGAACTCAGGAGTCATTTGGTTTCAATAAAATTATCAGCTTTC	Tessema et al., 2020	1000	10	0.227
t96	Forward	GTGACCTATGAACTCAGGAGTCTTTTCTCCACTTTGTAATTTTTATTGTTG	Tessema et al., 2020	1000	10	0.227
t98	Forward	GTGACCTATGAACTCAGGAGTCGATCTTCTCTGAAATTTACTTGAATTG	Tessema et al., 2020	1000	10	0.227
t99	Forward	GTGACCTATGAACTCAGGAGTCGATTATTTGGTAATGAACAACCAGG	Tessema et al., 2020	1000	10	0.227
ama1_D2_18	Reverse	CTGAGACTTGCACATCGCAGCCCTGCATGTCTTGAACATAAAGTC	This study	1000	10	0.227
cpmp_22	Reverse	CTGAGACTTGCACATCGCAGCTAGAATACGTGCTTTATAAACAAAGAG	This study	1000	10	0.227
cpp_30	Reverse	CTGAGACTTGCACATCGCAGCATTACTACCTTTCAGCATATCCGA	This study	1000	10	0.227
csp_27	Reverse	CTGAGACTTGCACATCGCAGCGAGCCAGGCTTTATTCTAACTTG	This study	1000	10	0.227
msp7_1	Reverse	CTGAGACTTGCACATCGCAGCGAATTTAAAACGAAGTCTTCATATTC	This study	1000	10	0.227
t01	Reverse	CTGAGACTTGCACATCGCAGCTGTCAAGGTATATTAAAGTATGGTATC	Tessema et al., 2020	1000	10	0.227
t11	Reverse	CTGAGACTTGCACATCGCAGCAATATAGTACCAGAAAATGGAAGAATG	Tessema et al., 2020	1000	10	0.227
t17	Reverse	CTGAGACTTGCACATCGCAGCGAAACTCCTACTACCAATAATTTGAC	Tessema et al., 2020	1000	10	0.227
t25	Reverse	CTGAGACTTGCACATCGCAGCAATATAACATTTATCCTATTGCTTAAAGG	Tessema et al., 2020	1000	10	0.227
t26	Reverse	CTGAGACTTGCACATCGCAGCACATCGCTAACAGAAATAATTTGATAC	Tessema et al., 2020	1000	10	0.227
t28	Reverse	CTGAGACTTGCACATCGCAGCGTATTTGGTTTGTCAGGCAATTCG	Tessema et al., 2020	1000	10	0.227
t30	Reverse	CTGAGACTTGCACATCGCAGCCCAAACATACATCCTCTTTTTGTTC	Tessema et al., 2020	1000	10	0.227
t31	Reverse	CTGAGACTTGCACATCGCAGCGTAGCATGCTCAGATATTATGATG	Tessema et al., 2020	1000	10	0.227
t33	Reverse	CTGAGACTTGCACATCGCAGCTTGTAGAATATTCGTGTCCGTATAG	Tessema et al., 2020	1000	10	0.227

t34	Reverse	CTGAGACTTGCACATCGCAGCGATGTAATTACATCCATACAATAACTTG	Tessema et al., 2020	1000	10	0.227
t35	Reverse	CTGAGACTTGCACATCGCAGCTTTCTTATTACATATGACACCACAAAC	Tessema et al., 2020	1000	10	0.227
t04	Reverse	CTGAGACTTGCACATCGCAGCGGAAAATCTTTGGTGGGAAAAATAG	Tessema et al., 2020	1000	10	0.227
t45	Reverse	CTGAGACTTGCACATCGCAGCAAAGTTTCTCTCTAAATTCATTCCAC	Tessema et al., 2020	1000	10	0.227
t47	Reverse	CTGAGACTTGCACATCGCAGCACTAAAAGGAAGACAAGCTGAAAAC	Tessema et al., 2020	1000	10	0.227
t49	Reverse	CTGAGACTTGCACATCGCAGCATTTATTACAACATTTTGATCATTCATGC	Tessema et al., 2020	1000	10	0.227
t53	Reverse	CTGAGACTTGCACATCGCAGCAGTTTTCTTCTGAATAGTTCTACAAAG	Tessema et al., 2020	1000	10	0.227
t54	Reverse	CTGAGACTTGCACATCGCAGCTTGTATATCCAAAATTACATTGACAATTG	Tessema et al., 2020	1000	10	0.227
t60	Reverse	CTGAGACTTGCACATCGCAGCCATCTATATAGTCCCCGATGAATG	Tessema et al., 2020	1000	10	0.227
t62	Reverse	CTGAGACTTGCACATCGCAGCAATGAGGACAAGGAAAATAATACATTC	Tessema et al., 2020	1000	10	0.227
t66	Reverse	CTGAGACTTGCACATCGCAGCATGAAACATATAAATTTGATTTCCAAGC	Tessema et al., 2020	1000	10	0.227
t67	Reverse	CTGAGACTTGCACATCGCAGCCTCCTTTAGGTATTACGGTAGC	Tessema et al., 2020	1000	10	0.227
t72	Reverse	CTGAGACTTGCACATCGCAGCACATTTATCTTATTTACCCGTATCTC	Tessema et al., 2020	1000	10	0.227
t73	Reverse	CTGAGACTTGCACATCGCAGCTCACCAACCTTTTTAGAATCAAGC	Tessema et al., 2020	1000	10	0.227
t74	Reverse	CTGAGACTTGCACATCGCAGCATCAACGACAAAAATAACAACTGATG	Tessema et al., 2020	1000	10	0.227
t08	Reverse	CTGAGACTTGCACATCGCAGCATATAAGAGATTCAAAGGGTCAGAC	Tessema et al., 2020	1000	10	0.227
t82	Reverse	CTGAGACTTGCACATCGCAGCGCTGGCGTATGTGTACACCT	Tessema et al., 2020	1000	10	0.227
t85	Reverse	CTGAGACTTGCACATCGCAGCAAGAATTTTAACACAAGGAGATCATC	Tessema et al., 2020	1000	10	0.227
t96	Reverse	CTGAGACTTGCACATCGCAGCGGGTGGTATCATGAGAATAGTTG	Tessema et al., 2020	1000	10	0.227
t98	Reverse	CTGAGACTTGCACATCGCAGCCAAACTTTTGGAGATTTCAAGATTATG	Tessema et al., 2020	1000	10	0.227
t99	Reverse	CTGAGACTTGCACATCGCAGCGGTCTTCCTAATCTTGAACCATC	Tessema et al., 2020	1000	10	0.227

Supplementary Table 15. Master mix composition for the primary multiplex ddPCR.

Reagents	Final concentration	Volume
NF ddH2O		6.5 µL
ddPCR Supermix for Probes (no dUTPs)	1X	11 µL
Primer Pool (35-plex)	227nM	0.5 µL
Template (DNA)		4 µL
Total		22 µL

Supplementary Table 16. Cycling conditions of multiplex ddPCR.

Step	Temperature	Time	Ramping	Cycles
Initial Denaturation	95°C	10 min	-	1
Denaturation	98°C	30 sec	2°C/s	25
Annealing/	58°C	2 min	2°C/s	
Extension				
Enzyme deactivation	98°C	10 min	-	1
Hold	4°C	∞	-	

Supplementary Table 17. PCR Primer sequences for AmpSeq library preparation (barcodes).

**Primer for Adapter PCR (XXXXXXXX=barcode)**	
Forward	AATGATACGGCGACCACCGAGATCTACACTCTTTCCCTACACGACGCTCTTCCGATCT**XXXXXXXX**GTGACCTATGAACTCAGGAGTC
Reverse	CAAGCAGAAGACGGCATACGAGATCGGTCTCGGCATTCCTGCTGAACCGCTCTTCCGATCT**XXXXXXXX**CTGAGACTTGCACATCGCAGC

**Forward barcode**		**Reverse barcode**	
F01	GCAACTGT	R01	TCCGATCT
F02	GAAGTACC	R02	ACGAGAGA
F03	GCCTTGAT	R03	ATCTGTCC
F04	CCAGGTTA	R04	TGCTGCAA
F05	GCTGACAA	R05	CCACTAAG
F06	TAGTGACG	R06	TACCTTGC
F07	GAGTTCGA	R07	CGATCACA
F08	AGAACCAC	R08	ACCAAGTG
F09	CTCCTCTA	R09	CGGAAGAA
F10	TTAACGCG	R10	AATGGTGG
F11	GGCATTCA	R11	GGTTCCTT
F12	CACTCTAG	R12	GGACATTG
F13	ATACAGGC	R13	TCATACGG
F14	AGTCGATC	R14	CGTTATGC
F15	TCTAGGAC	R15	CTGGCATT
F16	CACGAAGA	R16	ACTCTGCA
F17	AAGCACAG	R17	GAACGGAA
F18	AGCTTAGG	R18	TTGGTCAC
F19	CATAGCCA	R19	GTGAACCT
F20	TGGCAAGT	R20	ATCGCGAA
F21	GTCAGAAG	R21	TGAGTGGA
F22	GTTCCAGA	R22	CTGATTGG
F23	TCGCCTAA	R23	GTTGTGTG
F24	TGTGCAAG	R24	GCGTAATC

Supplementary Table 18. ENA accession numbers for WGS data from 242 samples from MalariaGEN[2] (Fws > 0.95) for relatedness inference and PCA/DAPC analyses.

SampleID	Country	run_accessions
PR0114-C	Bangladesh	ERR216644
PR0118-C	Bangladesh	ERR216532
PR0128-C	Bangladesh	ERR426105
PR0131-C	Bangladesh	ERR404251
PR0132-C	Bangladesh	ERR404211
PR0141-C	Bangladesh	ERR404237
PR0155-C	Bangladesh	ERR404241
PR0159-C	Bangladesh	ERR404190
PR0160-C	Bangladesh	ERR404230
PR0163-C	Bangladesh	ERR404203
PH1395-C	Cambodia	ERR905479
PH1396-C	Cambodia	ERR905480
PH1453-C	Cambodia	ERR1172522
PH1492-C	Cambodia	ERR1172518
PH1499-C	Cambodia	ERR1172559
PH1578-C	Cambodia	ERR1172579
PH1611-C	Cambodia	ERR1172534
PH1614-C	Cambodia	ERR1172535
PH1634-C	Cambodia	ERR1172586
QG0307-C	DRC	ERR1514617
QG0312-C	DRC	ERR1514619
QG0325-C	DRC	ERR1514622
QG0348-C	DRC	ERR1514555
QG0357-C	DRC	ERR1514564
QG0363-C	DRC	ERR1514570
QG0380-C	DRC	ERR1514576
QG0400-C	DRC	ERR1514583
QG0430-C	DRC	ERR1514600
QG0435-C	DRC	ERR1514602
PA0371-C	Gambia	ERR1106508
PA0384-C	Gambia	ERR1106519
PA0387-C	Gambia	ERR1106520
PA0396-C	Gambia	ERR1106528
PA0398-C	Gambia	ERR1106530
PA0400-C	Gambia	ERR1106506
PA0401-C	Gambia	ERR1106507
PA0417-C	Gambia	ERR1106543
PA0618-C	Gambia	ERR1138790
PA0655-C	Gambia	ERR1172503
PF1027-C	Ghana	ERR636070
PF1028-C	Ghana	ERR636077
PF1031-C	Ghana	ERR637303
PF1037-C	Ghana	ERR636060
PF1041-C	Ghana	ERR636086
PF1060-C	Ghana	ERR636054
PF1063-C	Ghana	ERR636072
PF1069-C	Ghana	ERR636033
PF1086-C	Ghana	ERR636068
PF1102-C	Ghana	ERR636098
PA0144-C	Guinea	ERR063550
PA0151-C	Guinea	ERR055482
PA0152-C	Guinea	ERR055493
PA0167-C	Guinea	ERR059407
PA0180-C	Guinea	ERR063529
PA0189-C	Guinea	ERR059411

PA0200-C	Guinea	ERR059396
PA0207-C	Guinea	ERR059397
PA0208-C	Guinea	ERR059387
PC0003-02	Kenya	ERR012359,ERR012498,ERR012257,ERR012334
PC0004-01	Kenya	ERR012380,ERR012226,ERR012509
PC0005-Cx	Kenya	ERR199976
PC0007-C	Kenya	ERR012277,ERR012237,ERR012228
PC0009-C	Kenya	ERR012409
PC0011-C	Kenya	ERR012475,ERR012330,ERR012446
PC0019-C	Kenya	ERR012227
PC0023-C	Kenya	ERR012317
PC0026-C	Kenya	ERR012519
PC0031-C	Kenya	ERR012425
PC0032-C	Kenya	ERR012494
PC0033-C	Kenya	ERR012304
PC0044-C	Kenya	ERR029094
PC0046-C	Kenya	ERR029095
PC0047-C	Kenya	ERR012481
PC0051-C	Kenya	ERR012456,ERR012489
PC0053-C	Kenya	ERR126559
PC0054-C	Kenya	ERR029083
PC0057-C	Kenya	ERR029085
PC0063-C	Kenya	ERR012752,ERR012478
PC0064-C	Kenya	ERR012428
PC0066-C	Kenya	ERR223047
PC0067-C	Kenya	ERR223039
PC0068-C	Kenya	ERR012381,ERR012518
PC0069-C	Kenya	ERR029413
PC0070-C	Kenya	ERR205934
PC0073-C	Kenya	ERR029414
PC0075-C	Kenya	ERR029412
PC0077-C	Kenya	ERR029088
PC0079-C	Kenya	ERR029104
PC0097-C	Kenya	ERR205958
PC0103-C	Kenya	ERR205959
PC0104-C	Kenya	ERR205951
PC0107-C	Kenya	ERR205952
PC0122-C	Kenya	ERR205955
QE0400-C	Laos	ERR180058
QE0405-Cx	Laos	ERR404175
QE0419-C	Laos	ERR223057
QE0430-C	Laos	ERR223075
QE0438-C	Laos	ERR223081
QE0440-C	Laos	ERR223083
QE0454-C	Laos	ERR216572
QE0455-C	Laos	ERR216573
QE0457-C	Laos	ERR216574
QE0475-C	Laos	ERR221487
PT0031-C	Malawi	ERR054070
PT0035-C	Malawi	ERR054071
PT0041-C	Malawi	ERR054074
PT0079-C	Malawi	ERR108409
PT0084-C	Malawi	ERR072008
PT0086-C	Malawi	ERR114394
PT0106-C	Malawi	ERR114390
PT0149-C	Malawi	ERR216485
PT0158-C	Malawi	ERR216580
PT0170-C	Malawi	ERR216600
PM0379-C	Mali	ERR670357
PM0395-C	Mali	ERR662092

PM0423-C	Mali	ERR670379
PM0435-C	Mali	ERR662085
PM0459-C	Mali	ERR670380
PM0478-C	Mali	ERR666929
PM0480-C	Mali	ERR666898
PM0494-C	Mali	ERR666861
PM0495-C	Mali	ERR670382
PM0510-C	Mali	ERR666905
QC0276-C	Myanmar	ERR1274866
QC0277-C	Myanmar	ERR1274867
QC0281-C	Myanmar	ERR1274870
QC0285-C	Myanmar	ERR1274874
QC0307-C	Myanmar	ERR1274889
QC0328-C	Myanmar	ERR1274905
QC0332-C	Myanmar	ERR1215361
QC0342-C	Myanmar	ERR1215371
QC0349-C	Myanmar	ERR1215378
QC0351-C	Myanmar	ERR1215380
QJ0007-C	Nigeria	ERR1172589
QJ0008-C	Nigeria	ERR1172590
QJ0018-C	Nigeria	ERR1172594
QJ0160-C	Nigeria	ERR1172604
QJ0164-C	Nigeria	ERR1172608
QJ0167-C	Nigeria	ERR1172609
QJ0169-C	Nigeria	ERR1172611
QJ0172-C	Nigeria	ERR1172615
QJ0174-C	Nigeria	ERR1172617
QJ0175-C	Nigeria	ERR1214226
PE0009-CW	Tanzania	ERR171645
PE0010-C	Tanzania	ERR018914
PE0011-C	Tanzania	ERR018915
PE0012-C	Tanzania	ERR018916
PE0013-C	Tanzania	ERR022906
PE0018-C	Tanzania	ERR027092
PE0019-C	Tanzania	ERR022907
PE0020-C	Tanzania	ERR022920
PE0021-C	Tanzania	ERR022908
PE0022-C	Tanzania	ERR022531
PE0024-C	Tanzania	ERR022909
PE0028-C	Tanzania	ERR171647
PE0087-C	Tanzania	ERR405240
PE0089-C	Tanzania	ERR405243
PE0090-C	Tanzania	ERR405245
PE0105-C	Tanzania	ERR405252
PE0113-C	Tanzania	ERR405244
PE0123-C	Tanzania	ERR439525
PE0124-C	Tanzania	ERR439528
PE0125-C	Tanzania	ERR439531
PE0126-C	Tanzania	ERR449895
PE0130-C	Tanzania	ERR449903
PE0131-C	Tanzania	ERR449905
PE0133-C	Tanzania	ERR439520
PE0134-C	Tanzania	ERR439523
PE0135-C	Tanzania	ERR439526
PE0137-C	Tanzania	ERR439532
PE0138-C	Tanzania	ERR449896
PE0141-C	Tanzania	ERR449902
PE0147-C	Tanzania	ERR439527
PE0151-C	Tanzania	ERR689149
PE0161-C	Tanzania	ERR689211

PE0164-C	Tanzania	ERR696953
PE0172-C	Tanzania	ERR689223
PE0173-C	Tanzania	ERR689186
PE0179-C	Tanzania	ERR689210
PE0184-C	Tanzania	ERR689224
PE0197-C	Tanzania	ERR689153
PE0199-C	Tanzania	ERR689174
PE0201-C	Tanzania	ERR689151
PE0202-C	Tanzania	ERR689152
PE0207-C	Tanzania	ERR676491
PE0213-C	Tanzania	ERR689161
PE0254-C	Tanzania	ERR689212
PE0255-C	Tanzania	ERR676497
PE0258-C	Tanzania	ERR676493
PE0273-C	Tanzania	ERR676472
PE0278-C	Tanzania	ERR676484
PE0282-C	Tanzania	ERR676504
PE0294-C	Tanzania	ERR676482
PE0301-C	Tanzania	ERR689190
PE0303-C	Tanzania	ERR689191
PE0305-C	Tanzania	ERR676469
PE0307-C	Tanzania	ERR676495
PE0314-C	Tanzania	ERR676485
PE0330-C	Tanzania	ERR676494
PE0335-C	Tanzania	ERR689189
PE0336-C	Tanzania	ERR676508
PE0337-C	Tanzania	ERR676510
PE0341-C	Tanzania	ERR689167
PE0349-C	Tanzania	ERR696961
PE0350-C	Tanzania	ERR689178
PE0362-C	Tanzania	ERR696945
PE0366-C	Tanzania	ERR676511
PE0385-C	Tanzania	ERR696949
PE0390-C	Tanzania	ERR689156
PE0393-C	Tanzania	ERR676499
PE0406-C	Tanzania	ERR689205
PE0411-C	Tanzania	ERR676498
PE0412-C	Tanzania	ERR689228
PE0416-C	Tanzania	ERR676522
PE0424-C	Tanzania	ERR689166
PE0425-C	Tanzania	ERR689179
PE0426-C	Tanzania	ERR689180
PE0430-C	Tanzania	ERR676486
PE0431-C	Tanzania	ERR676487
PE0436-C	Tanzania	ERR689207
PE0437-C	Tanzania	ERR689171
PE0440-C	Tanzania	ERR689182
PE0447-C	Tanzania	ERR676501
PD0462-C	Thailand	ERR164726
PD0521-C	Thailand	ERR216506
PD0824-C	Thailand	ERR580376
PD1161-C	Thailand	ERR596140
PD1163-C	Thailand	ERR596151
PD1189-C	Thailand	ERR689288
PD1191-C	Thailand	ERR689264
PD1204-C	Thailand	ERR689254
PD1220-C	Thailand	ERR689327
PD1223-C	Thailand	ERR689308
PV0241-C	Vietnam	ERR126570
PV0249-Cx	Vietnam	ERR388792

PV0265-C	Vietnam	ERR171582
PV0280-C	Vietnam	ERR180089
PV0301-C	Vietnam	ERR164737
PV0304-C	Vietnam	ERR216543
PV0315-Cx	Vietnam	ERR404180
PV0326-C	Vietnam	ERR221523
PV0331-Cx	Vietnam	ERR404181

SUPPLEMENTARY FIGURES

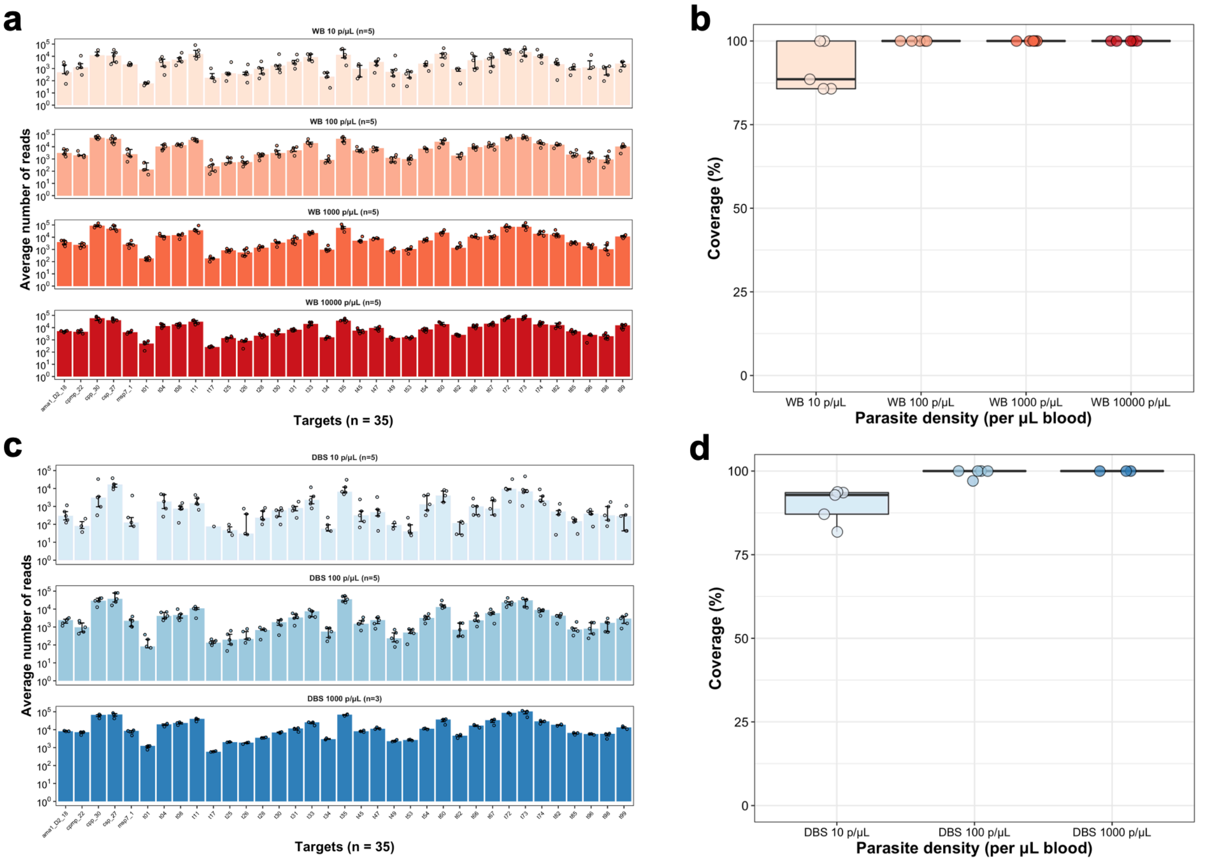

Supplementary Figure 1. Evenness and coverage of multiplexed amplicon sequencing of microhaplotypes (*n*=28) and drug resistance loci (*n*=7). **A & C**, Average number of reads per target per sample. The median (bars) and interquartile range (error bars) are shown. Number of samples per marker is indicated. **B & D**, Boxplot summarizing the coverage of microhaplotype loci and drug resistance targets by parasite density. Coverage was determined based on the number of targets with 10 or more reads (e.g., 100% indicates 35/35 loci with ≥10 reads). The box bounds the IQR divided by the median, and Tukey-style whiskers extend to a maximum of 1.5 × IQR beyond the box. Samples are 3D7 whole blood (reds) ranging from 10,000 – 10 parasites/μL (*n*=5 for all densities) or 3D7 DBS (blues) ranging from 1,000 – 10 parasites/μL (*n*=3 for 1,000 parasites/μL; *n*=5 for 100 and 10 parasites/μL).

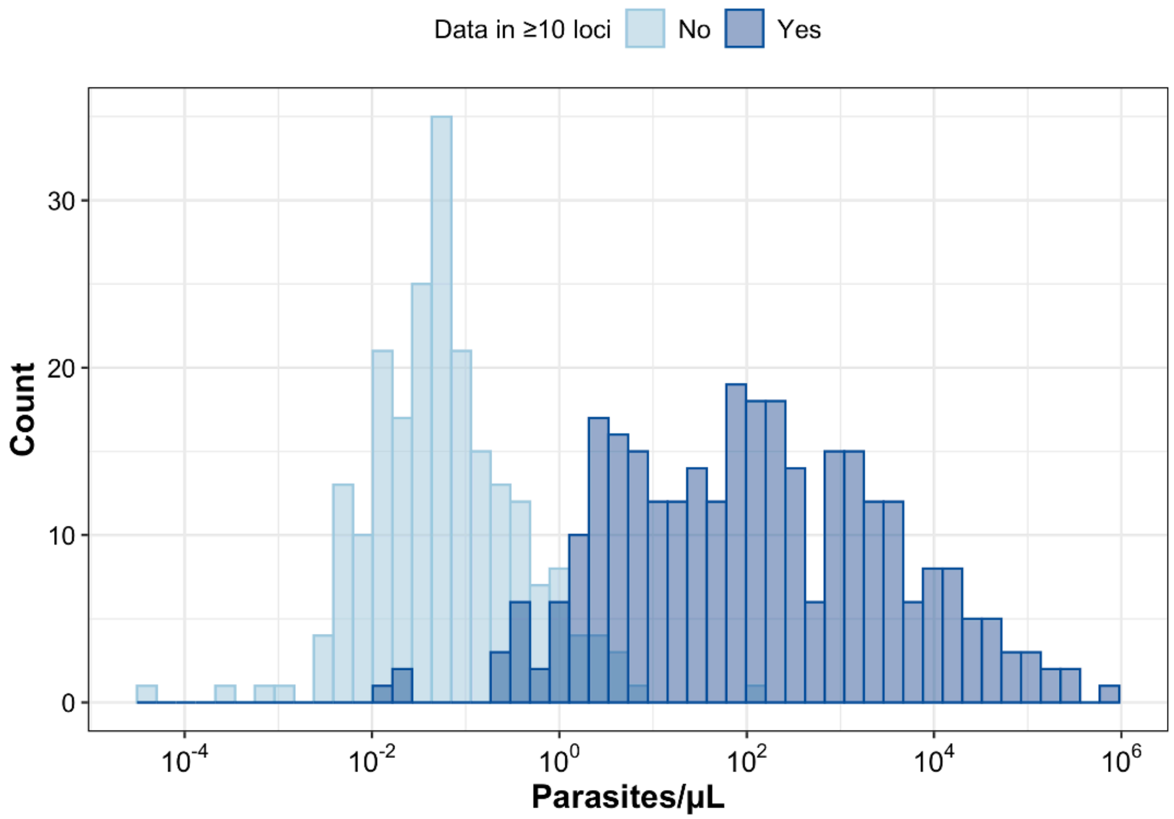

Supplementary Figure 2. Distribution of parasite densities of the 518 DBS samples from Zanzibar. Samples that were included for further analysis (data in ≥10 loci, *n*=290) are indicated.

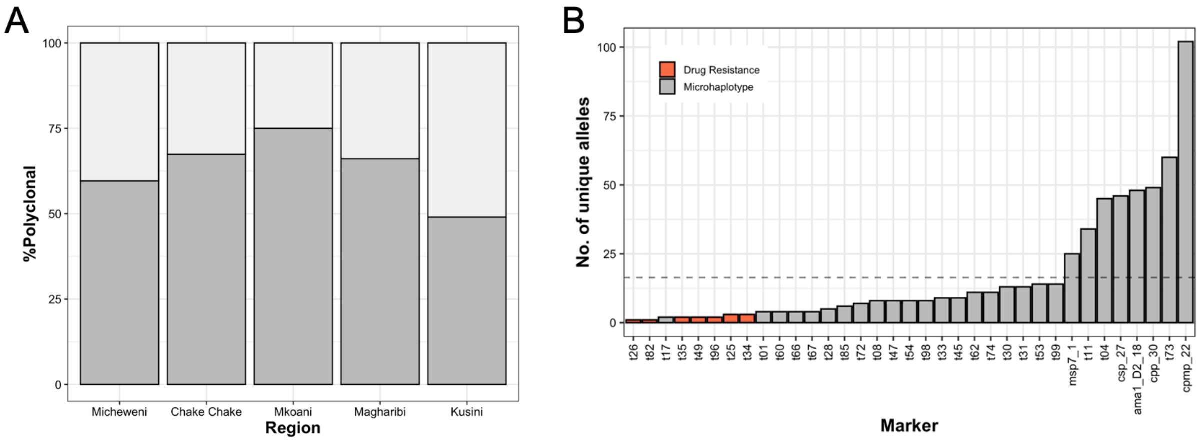

Supplementary Figure 3. A, Percentage polyclonal infections by the 5 districts in Zanzibar. ($X^2(16) = 6.49$, $P = 0.166$). *P* value was two-tailed and computed using the Pearson's Chi-squared test. **B,** Number of unique alleles of microhaplotypes and drug resistance loci in all 290 Zanzibar samples (mean A: 16.7, range: 1-102). Dashed line indicates mean A.

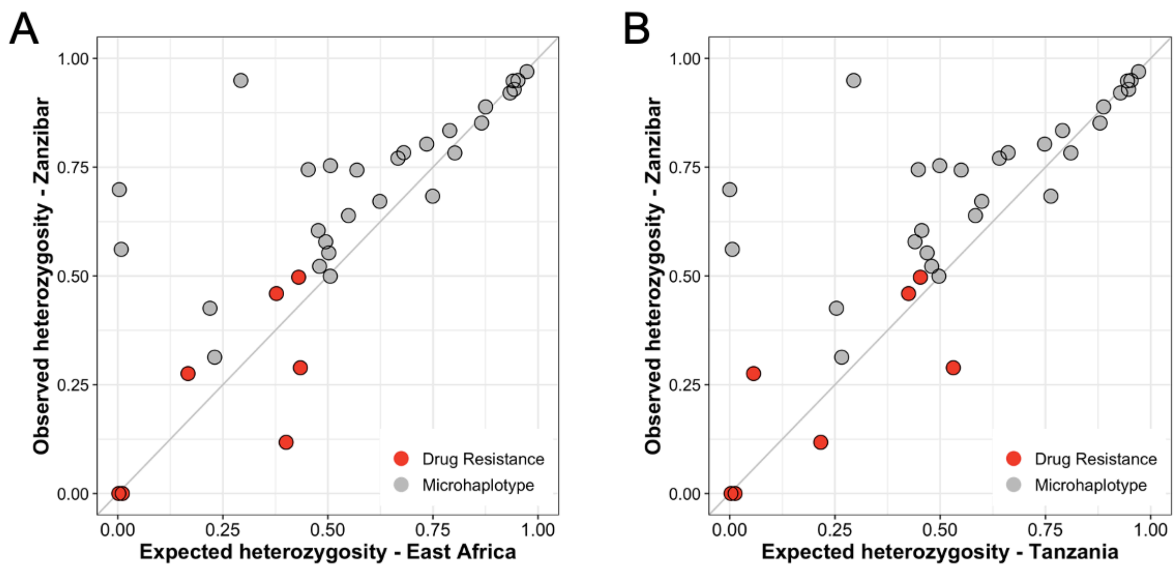

Supplementary Figure 4. A, Correspondence between expected heterozygosity of microhaplotypes and drug resistance loci between samples from East Africa and the observed heterozygosity in the Zanzibar samples was assessed by simple linear regression (R^2 = 0.57, F(1, 33) = 44.52, P = 1.3e-7). **B**, Correspondence between expected heterozygosity of microhaplotypes and drug resistance loci between samples from Tanzania and the observed heterozygosity in the Zanzibar samples was assessed by simple linear regression (R^2 = 0.59, F(1, 33) = 47.38, P = 7.4e-8).

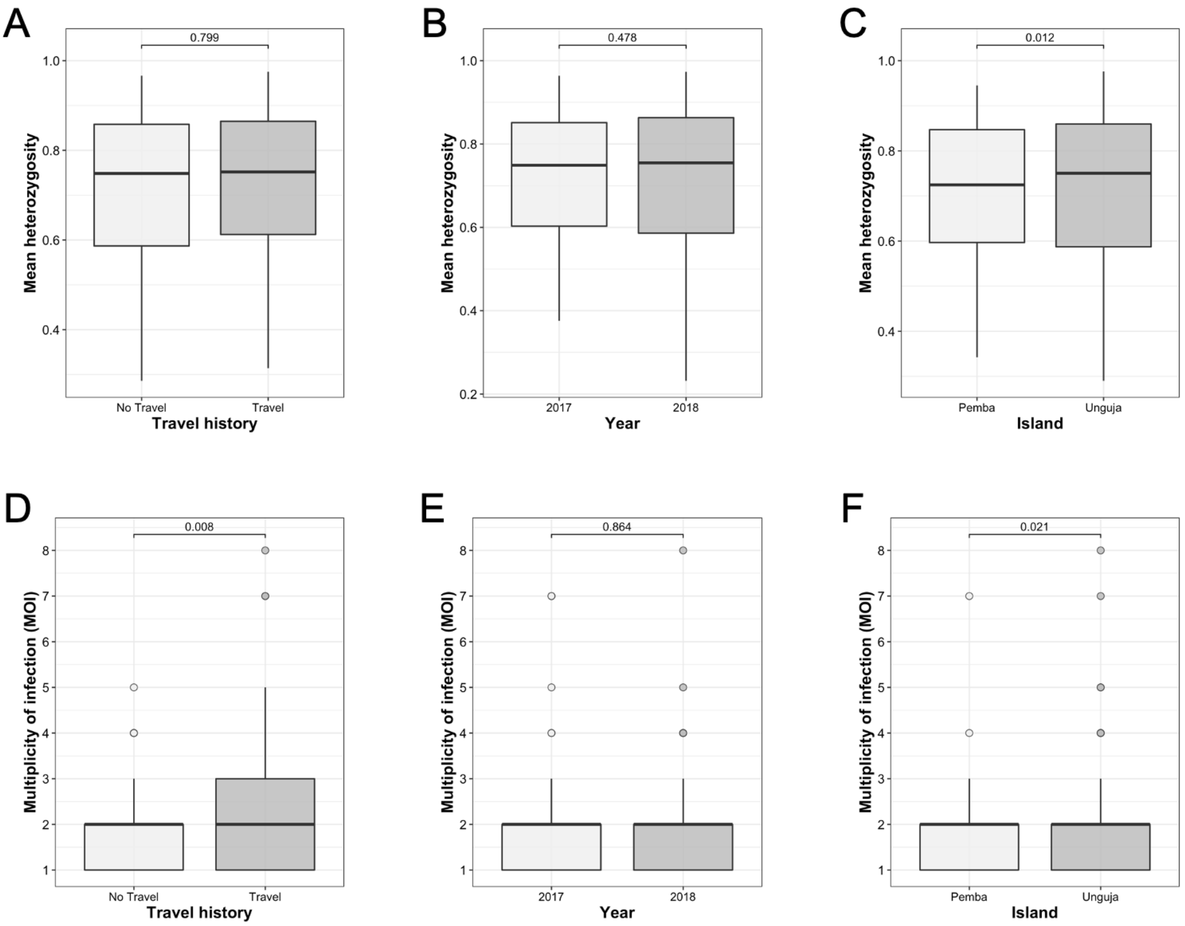

Supplementary Figure 5. Multiplicity of infection and population diversity by travel history, year, and island. **A, B & C**, Population diversity measured as the distribution of heterozygosity in microhaplotypes (*n*=28 for each comparison). Differences were examined using a pairwise two-sided t-test. Bonferroni adjusted pairwise *P* values are indicated. **D, E & F**, Multiplicity of infection (MOI) by travel history (no travel *n*=206; travel *n*=84), year (2017 *n*=154; 2018 *n*=136), and island (Pemba *n*=120; Unguja *n*=170). Differences were examined using a two-sided t-test Bonferroni adjusted *P* values are indicated. For all box plots the box bounds the IQR divided by the median, and Tukey-style whiskers extend to a maximum of 1.5 × IQR beyond the box.

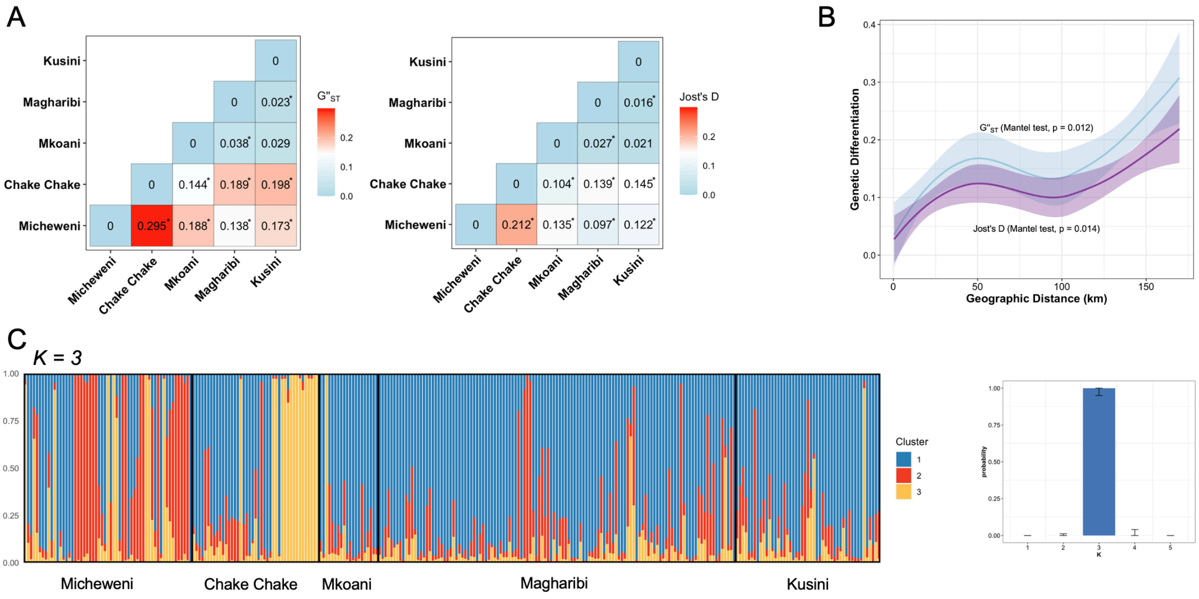

Supplementary Figure 6. Genetic differentiation and population structure. **A,** Pairwise G''_{ST} (left) and *Jost's D* (right) between districts. Differences between districts were examined by one-sided permutation test (using 100,000 permutations); *$P < 0.01$. P values for each comparison were G''_{ST} Micheweni vs. Chake Chake P < 1e-5, Micheweni vs. Mkoani P < 1e-5, Micheweni vs. Magharibi P < 1e-5, Micheweni vs. Kusini P < 1e-5, Chake Chake vs. Mkoani P = 0.005, Chake Chake vs. Magharibi P < 21e-5, Chake Chake vs. Kusini P < 1e-5, Mkoani vs. Magharibi P = 0.014, Magharibi vs. Kusini P = 0.006; Jost's D Micheweni vs. Chake Chake P < 1e-5, Micheweni vs. Mkoani P < 1e-5, Micheweni vs. Magharibi P < 1e-5, Micheweni vs. Kusini P < 1e-5, Chake Chake vs. Mkoani P = 0.004, Chake Chake vs. Magharibi P < 1e-5, Chake Chake vs. Kusini P < 1e-5, Mkoani vs. Magharibi P = 0.014, Magharibi vs. Kusini P = 0.005.**B**, Relationship between pairwise genetic differentiation and geographic distance in Zanzibar between 29 shehia pairs (n=406 pairs) with sample sizes ≥3 for G''_{ST} (Mantel statistic r = 0.246, P = 0.012), and for *Jost's D* (Mantel statistic r = 0.238, P = 0.014). The Mantel statistic is based on Spearman's rank correlation r, and significance was examined by one-sided permutation test (using 100,000 permutations). Error bands indicate 95% CI. **C,** Population cluster analysis of *P. falciparum* microhaplotypes from dominant alleles in Zanzibar. Individual ancestry coefficients (left) and optimum K value (K=3, right) are shown as inferred by *rmaverick*[3]. Each vertical bar represents an individual haplotype and its membership to the three cluster are defined by the different colors. Black borders separate the five districts. Error bars indicate 95% credible intervals.

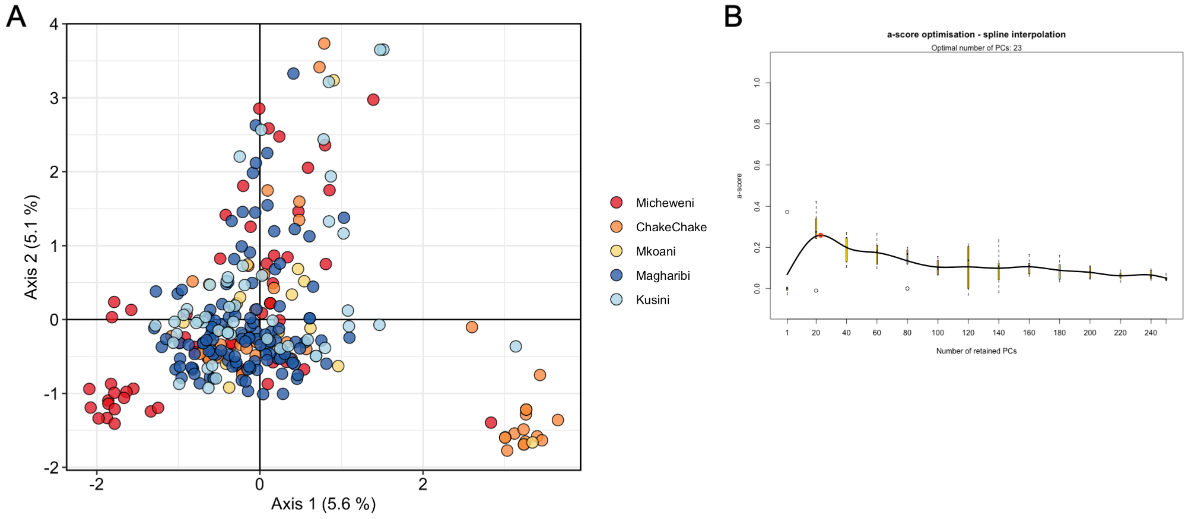

Supplementary Figure 7. A, Principal components analysis (PCA) using allele frequency of the 28 microhaplotypes. The first two PCA components are shown (explaining 5.6% and 5.1% of variation in the dataset, respectively). **B,** Ascertaining the number of components to include in the discriminatory analysis of principal components (DAPC). Alpha-score optimization shows the optimal number of principal components (23 PCs) to retain for DAPC analysis based on the maximum a-score (red dot). These 23 PCs explain 49.8% of variation in the dataset.

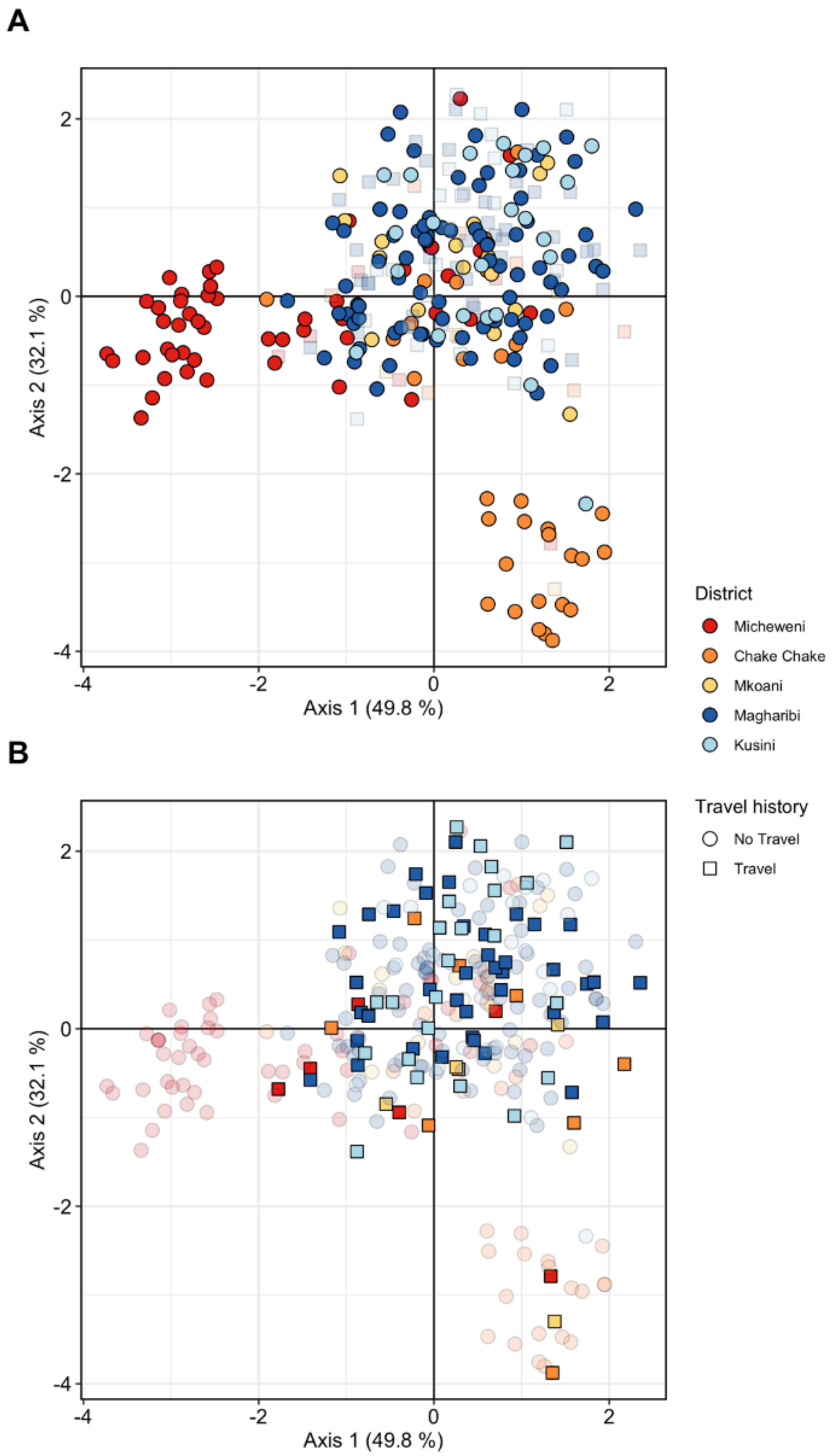

Supplementary Figure 8. Separation of *P. falciparum* samples by district using discriminatory analysis of principal components (DAPC). The first 23 components of a principal component analysis (PCA) are shown. **A**, highlighting non-travelers (*n*=206). **B**, highlighting travelers (*n*=84). Each dot represents a sample colored by its geographic origin based on the 5 districts included in the analysis.

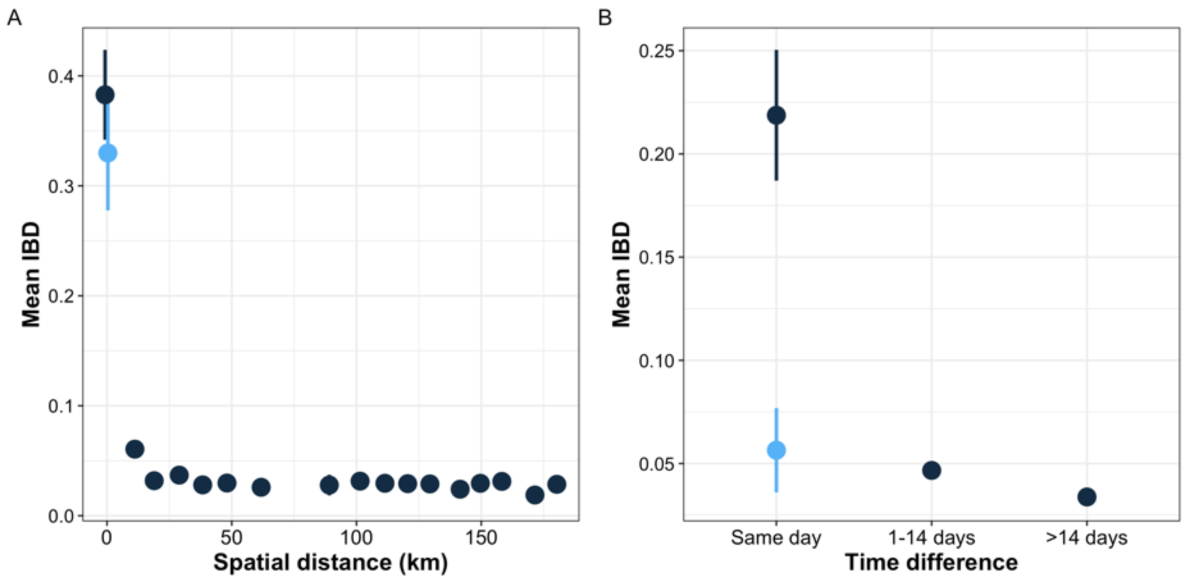

Supplementary Figure 9. Mean IBD by spatial distance and time difference. **A,** Spatial patterns in IBD among all samples (*n*=290). Mean IBD binned by the spatial distance in 10km intervals. Vertical lines indicate 95% confidence intervals. Blue dot indicates mean IBD where RACD pairs were excluded. **B,** Temporal patterns in IBD among all samples (*n*=290). Mean IBD of sample pairs collected on the same day, 1-14 days apart, or >14 days apart. Vertical lines indicate 95% confidence intervals. Blue dot indicates mean IBD where RACD pairs were excluded.

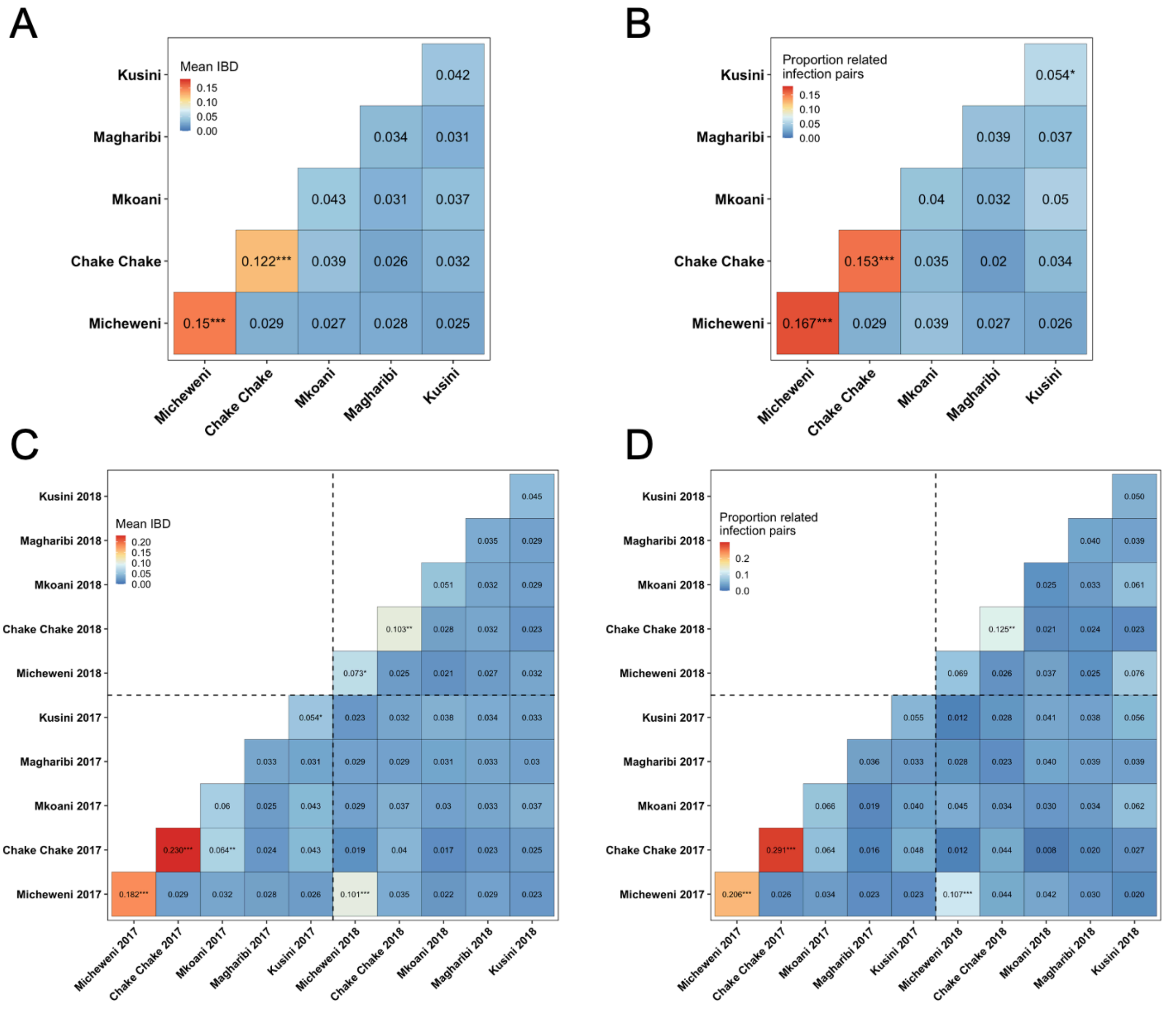

Supplementary Figure 10. A, Mean IBD by districts. Differences within/between districts were examined by one-sided permutation test (using 100,000 permutations); ****P* < 0.001. *P* values for each comparison were Micheweni vs. Micheweni *P* < 1e-5 and Chake Chake vs. Chake Chake *P* < 1e-5. **B,** Proportion of related infection pairs by district. Differences within/between districts were examined by one-sided permutation test (using 100,000 permutations); ****P* < 0.001. *P* values for each comparison were Micheweni vs. Micheweni *P* < 1e-5 and Chake Chake vs. Chake Chake *P* < 1e-5. **C,** Mean IBD by districts stratified by year. Differences within/between districts by year were examined by one-sided permutation test (using 100,000 permutations); **P* < 0.05, ***P* < 0.01, and ****P* < 0.001. *P* values for each comparison were Micheweni 2017 vs. Micheweni 2017 *P* < 1e-5, Micheweni 2017 vs. Micheweni 2018 *P* < 1e-5, Chake Chake 2017 vs. Chake Chake 2017 *P* < 1e-5, Chake Chake 2017 vs. Mkoani 2017 *P* = 0.004, Kusini 2017 vs. Kusini 2017 *P* = 0.034, Micheweni 2018 vs. Micheweni 2018 *P* = 0.027, Chake Chake 2018 vs. Chake Chake 2018 *P* = 0.001. **D,** Proportion of related infection pairs by district stratified by year. Differences within/between districts by year were examined by one-sided permutation test (using 100,000 permutations); ***P* < 0.01, and ****P* < 0.001. *P* values for each comparison were Micheweni 2017 vs. Micheweni 2017 *P* < 1e-5, Micheweni 2017 vs. Micheweni 2018 *P* < 1e-5, Chake Chake 2017 vs. Chake Chake 2017 *P* < 1e-5, Chake Chake 2018 vs. Chake Chake 2018 *P* = 0.001.

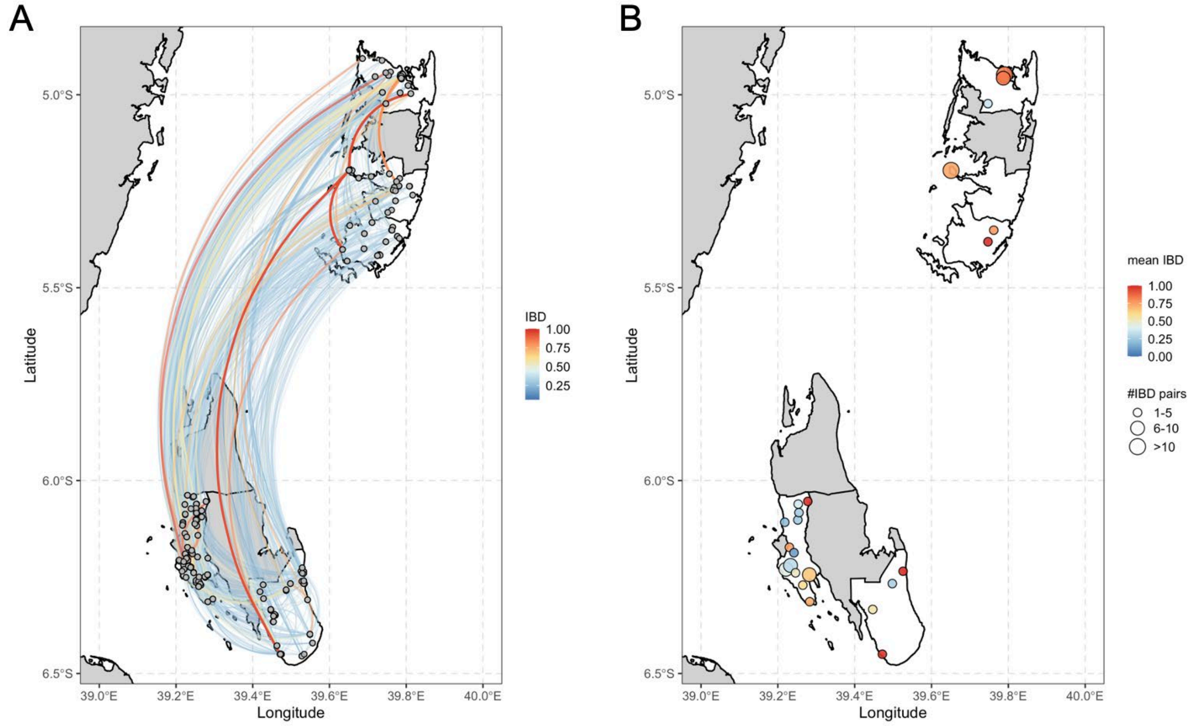

Supplementary Figure 11. All significantly related pairs (*n*=1,703) as determined by *dcifer*[4] (**A**) between shehia (*n*=1,335) and (**B**) within shehia (*n*=368). Within shehia mean IBD is higher on Pemba Island (0.78) than on Unguja Island (0.49).

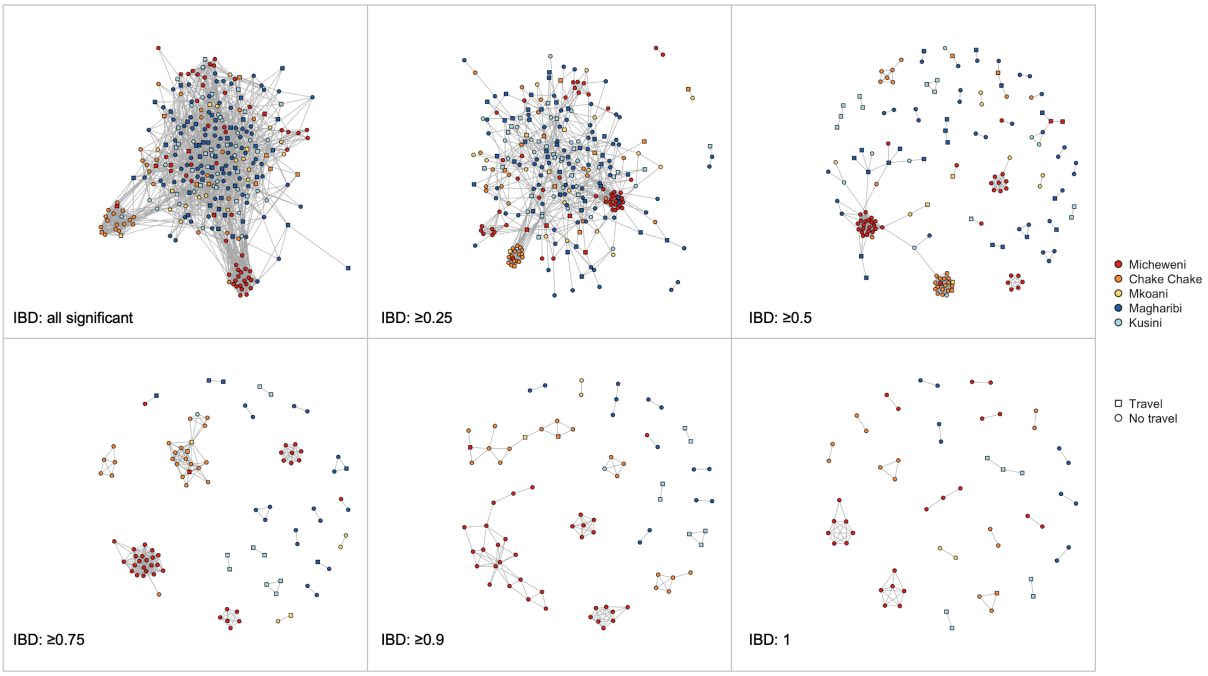

Supplementary Figure 12. Network analysis of pairwise relatedness visualized using different thresholds of IBD and colored by district. Isolates that share less than the indicated IBD threshold with any other isolate are omitted from the network.

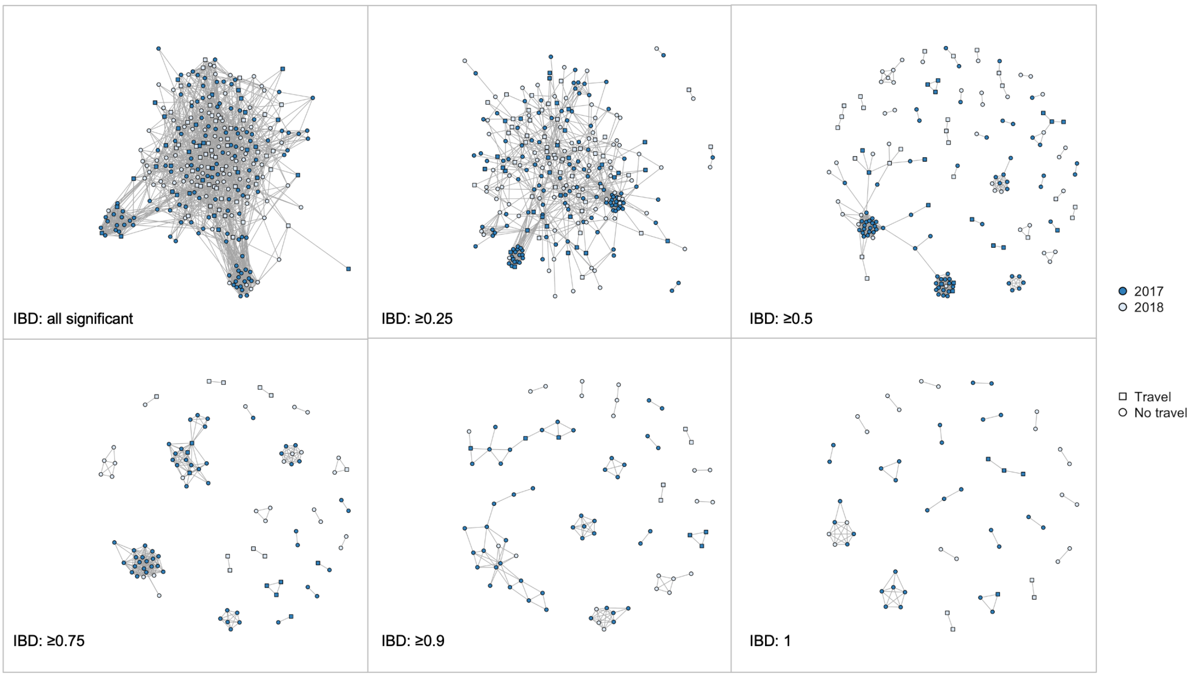

Supplementary Figure 13. Network analysis of pairwise relatedness visualized using different thresholds of IBD and colored by year. Isolates that share less than the indicated IBD threshold with any other isolate are omitted from the network.

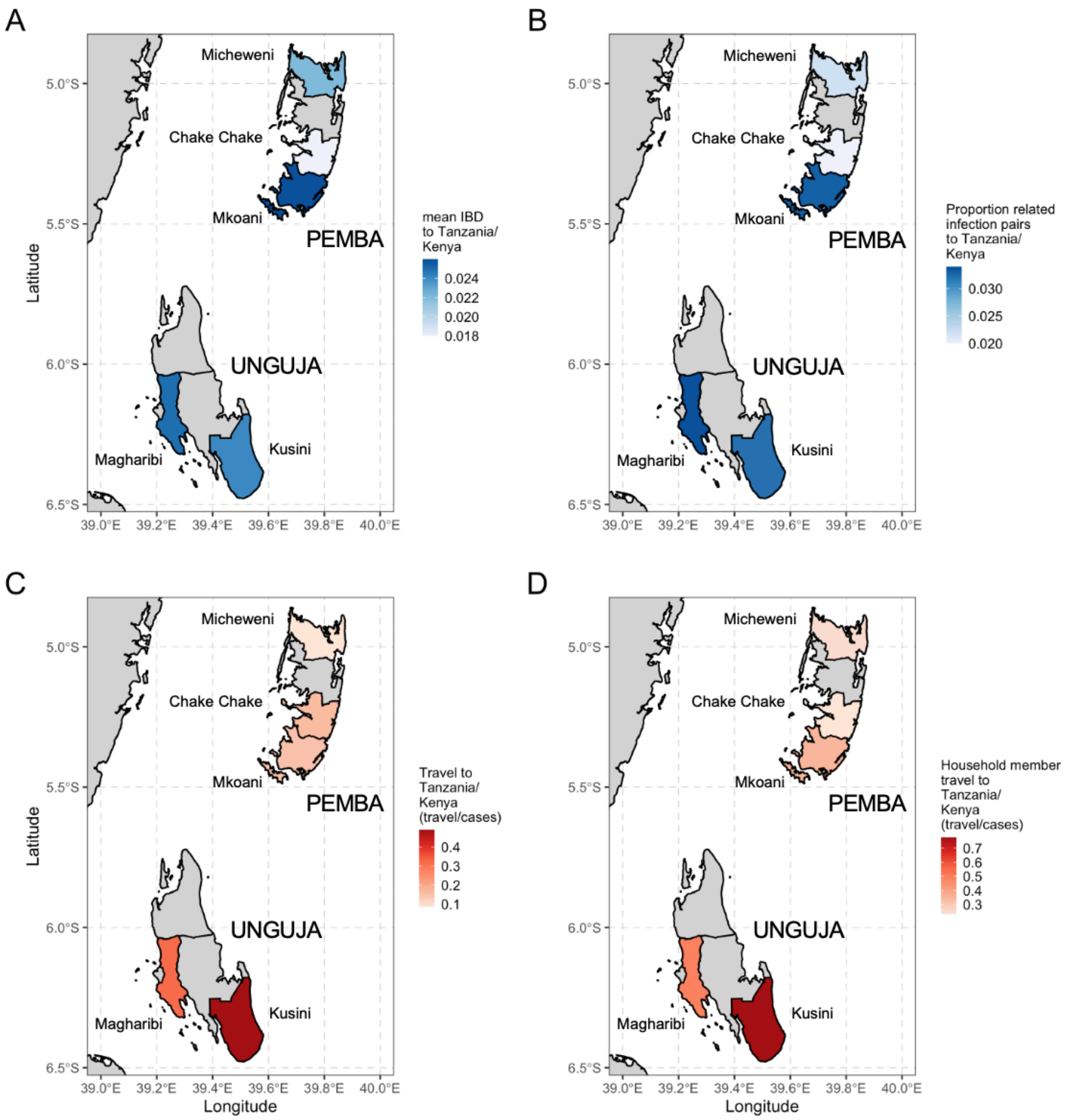

Supplementary Figure 14. Map showing mean IBD and proportion of related infection of the five districts to mainland Tanzania and Kenya. **A**, Mean IBD of Micheweni (0.022), Chake Chake (0.018), Mkoani (0.026), Magharibi (0.025) and Kusini (0.024) from north to south. **B,** Proportion of related infection of Micheweni (0.022), Chake Chake (0.020), Mkoani (0.033), Magharibi (0.034) and Kusini (0.032). **C**, Proportion of people with genotyping data that reported travel to the mainland: Micheweni (8.8%), Chake Chake (16.3%), Mkoani (15.0%), Magharibi (32.2%) and Kusini (49.0%) from north to south. **D**, Proportion of household members that reported travel to the mainland from those individuals with genotyping data: of Micheweni (24.6%), Chake Chake (23.3%), Mkoani (35.0%), Magharibi (49.6%) and Kusini (77.6%) from north to south.

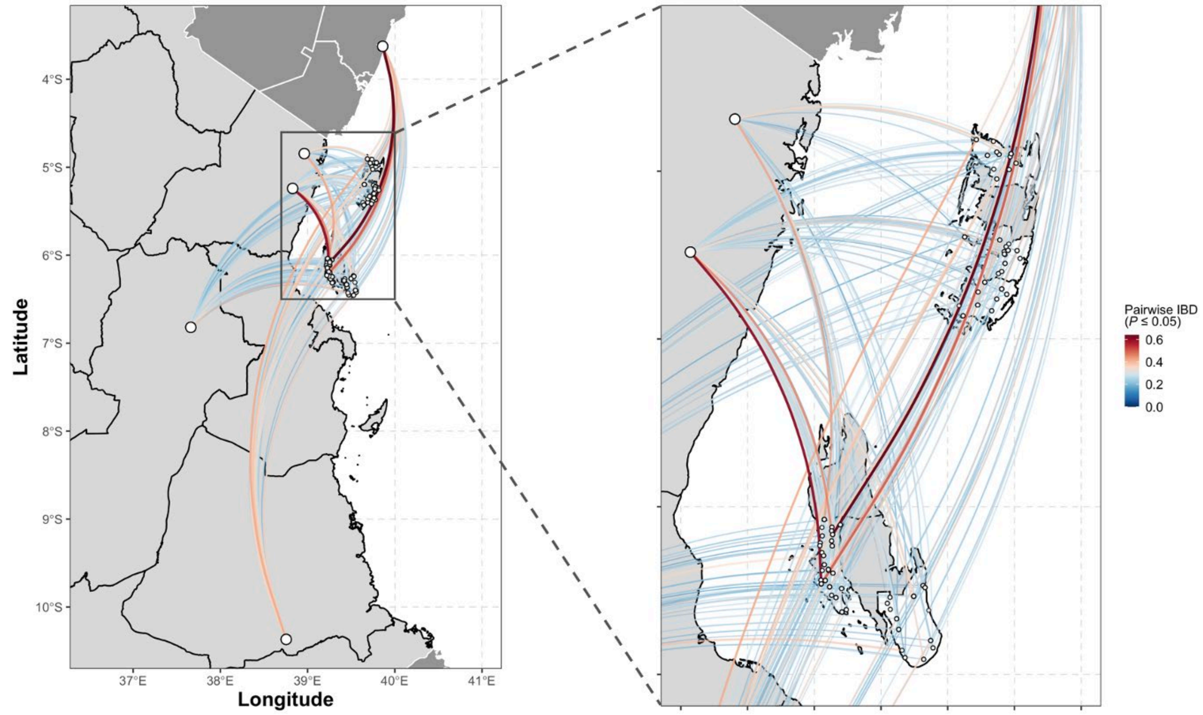

Supplementary Figure 15. Map with all significantly related infection pairs as determined by *dcifer*[4] (i.e., at a significance level of $P \leq 0.05$, n=976) between samples from Zanzibar and locations in mainland Tanzania (n=4) and Kenya (n=1). Three infection pairs with IBD >0.5 were identified.

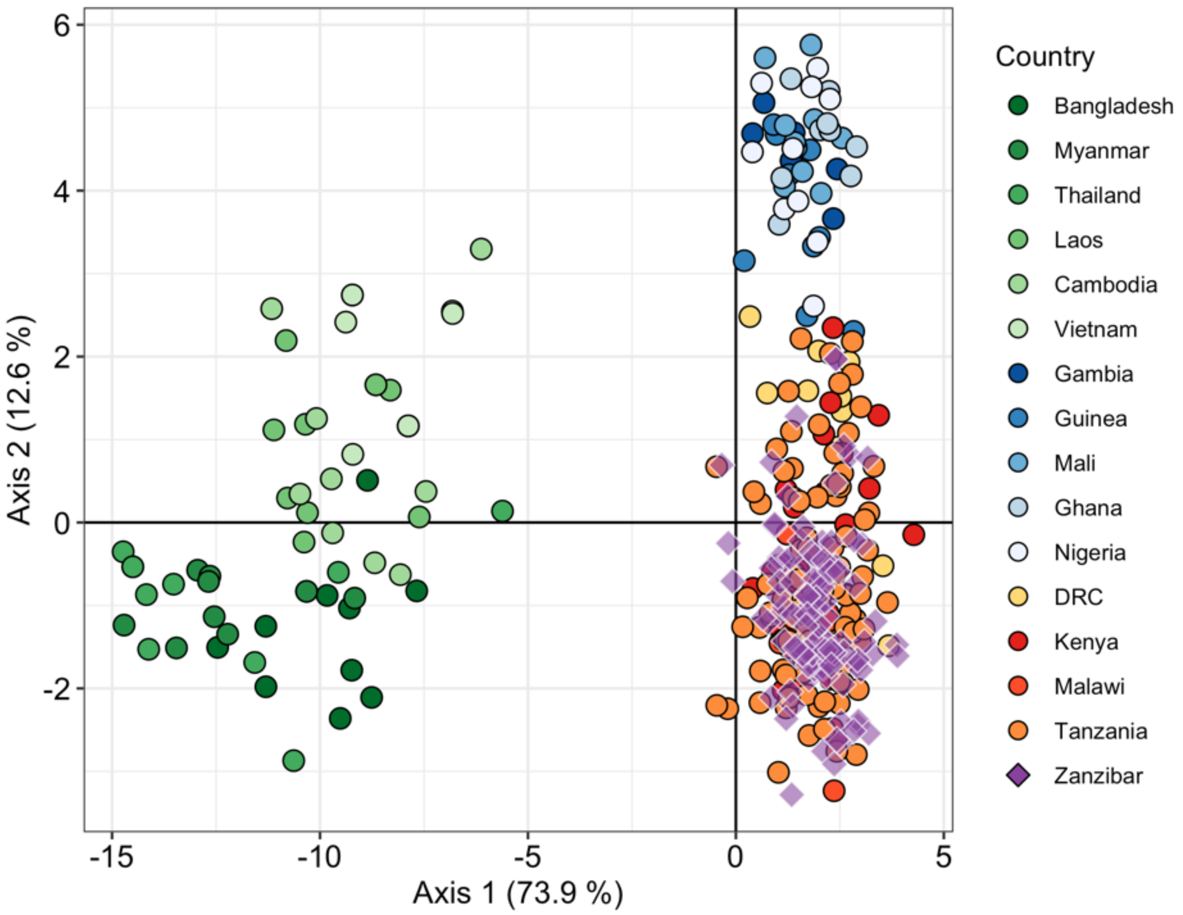

Supplementary Figure 16. Genetic differentiation between global *P. falciparum* populations by discriminatory analysis of principal components (DAPC) using 28 microhaplotypes and 7 drug resistance loci. The first 48 components of a principal component analysis (PCA) are shown. Each point represents a single isolate (*n*=359); colors indicate country of origin. Only monoclonal samples were used for this analysis.

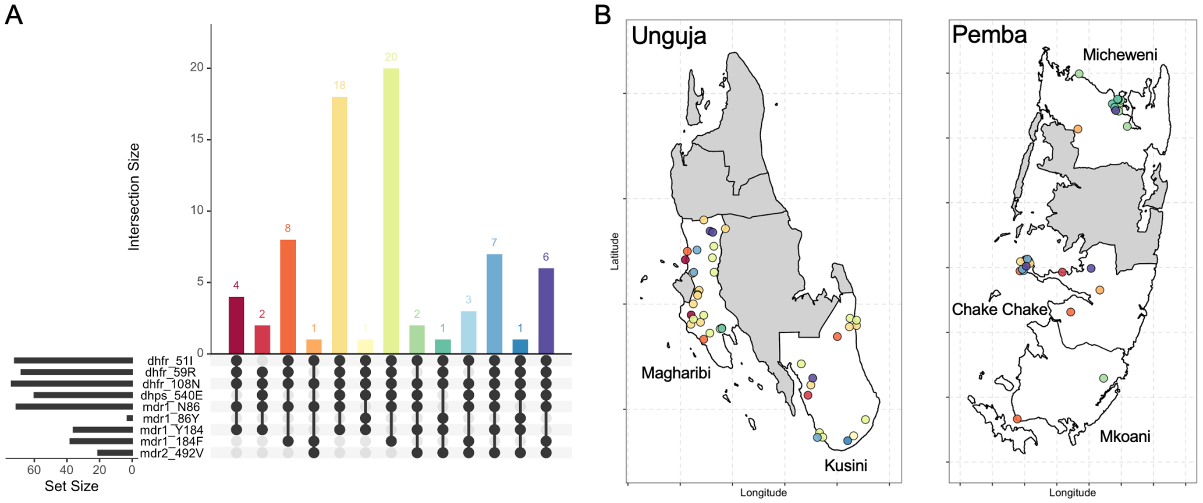

Figure 17. The spatial distribution of all combinations of multi-resistant isolates from monoclonal Zanzibar samples with complete allele calls (*n*=74). **A,** UpSetR[5] plot showing the number of isolates carrying different combinations of antimalarial drug resistance-associated alleles in four genes (*pfdhfr, pfdhps, pfmdr1*, and *pfmdr2*). **B,** Map showing drug resistant isolates on Unguja (left) and Pemba (right). Colors correspond between panels.

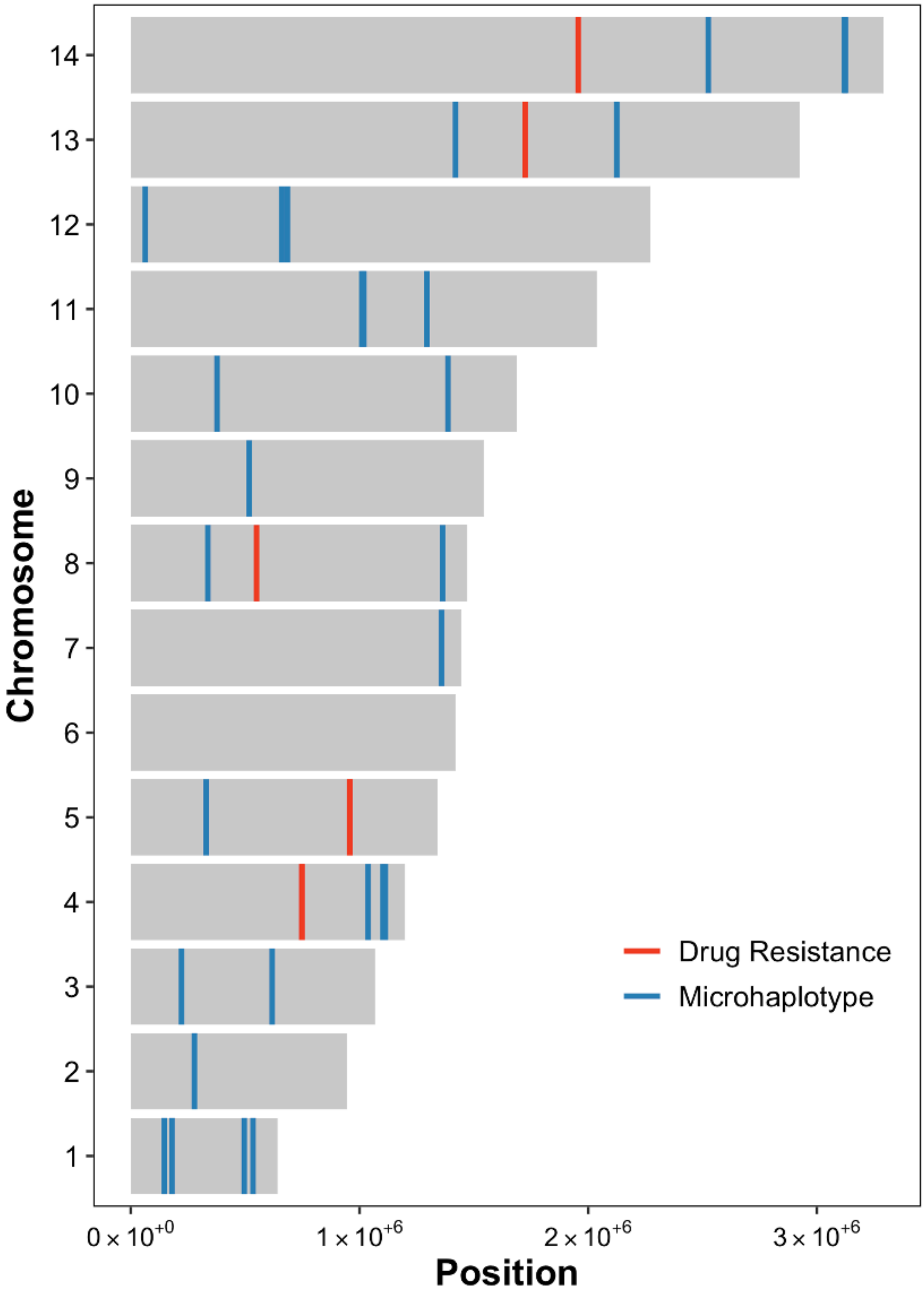

Supplementary Figure 18. Chromosomal location of microhaplotypes (blue) and drug resistance loci (red) in the *P. falciparum* genome.

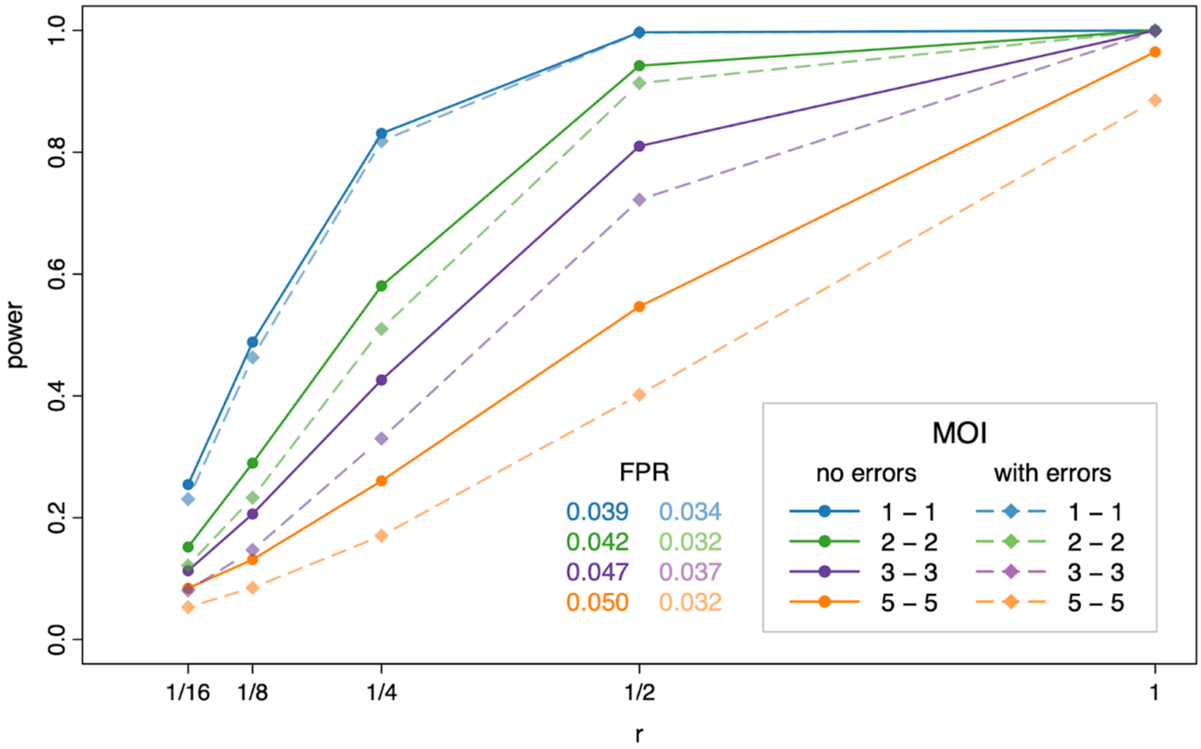

Supplementary Figure 19. Comparison of hypothesis testing results for *Dcifer*, using likelihood-ratio *P* values adjusted for one-sided tests at a significance level α=0.05 without adjustments for multiple testing. Two simulation schemes described elsewhere[4] were used: (a) no genotyping errors; true MOI and population allele frequencies used as inputs to *dcifer*, and (b) including genotyping error with fixed error model parameters; MOIs and allele frequencies estimated from these data. False-positivity rate (FPR) was below the nominal significance level α=0.05 across different simulations of MOI. Statistical power was greater for higher values of relatedness and higher MOI led to lower power. For our panel of 28 microhaplotypes, the power to detect siblings (r = 0.5) in a pair of infections with MOI of 2 was 0.91, and 0.72 in a pair of infections with MOI of 3; in our study MOI between most infection pairs was rarely above 2-3.

REFERENCES

1. Haubold, B. & Hudson, R. R. LIAN 3.0: detecting linkage disequilibrium in multilocus data. *Bioinformatics* **16**, 847–849 (2000).
2. MalariaGEN *et al.* Pf7: an open dataset of Plasmodium falciparum genome variation in 20,000 worldwide samples. *Wellcome Open Res* **8**, 22 (2023).
3. Verity, R. & Nichols, R. A. Estimating the Number of Subpopulations (K) in Structured Populations. *Genetics* **203**, 1827–1839 (2016).
4. Gerlovina, I., Gerlovin, B., Rodríguez-Barraquer, I. & Greenhouse, B. Dcifer: an IBD-based method to calculate genetic distance between polyclonal infections. 2022.04.14.488406 Preprint at https://doi.org/10.1101/2022.04.14.488406 (2022).
5. Lex, A., Gehlenborg, N., Strobelt, H., Vuillemot, R. & Pfister, H. UpSet: Visualization of Intersecting Sets. *IEEE Transactions on Visualization and Computer Graphics* **20**, 1983–1992 (2014).
