# Supplementary material for: Multiplexed ddPCR-amplicon sequencing reveals isolated Plasmodium falciparum populations amenable to local elimination in Zanzibar, Tanzania
Source: Nat Commun. 2023 Jun 22;14:3699. doi: 10.1038/s41467-023-39417-1 (PMC10287761; doi:10.1038/s41467-023-39417-1)
Supplement: Supplementary file 3 — Description of Additional Supplementary Files [file 41467_2023_39417_MOESM3_ESM.pdf]

Description of Additional Supplementary Files

File Name: Supplementary Data 1
Description: Epidemiological data of all 290 samples included in the analyses. Information about date of collection, travel history, symptoms, index case vs secondary case, RDT result, household ID, and origin.

File Name: Supplementary Data 2
Description: Haplotype calls for the 35 loci of all 290 samples included in the analyses.

File Name: Supplementary Data 3
Description: Illumina barcode IDs of all samples.

File Name: Supplementary Data 4
Description: Haplotype calls for the 28 microhaplotype loci of 242 publicly available WGS isolates from MalariaGEN.
