# Supplementary material for: Multiplexed ddPCR-amplicon sequencing reveals isolated Plasmodium falciparum populations amenable to local elimination in Zanzibar, Tanzania
Source: Nat Commun. 2023 Jun 22;14:3699. doi: 10.1038/s41467-023-39417-1 (PMC10287761; doi:10.1038/s41467-023-39417-1)
Supplement: Supplementary file 8 — Reporting Summary [file 41467_2023_39417_MOESM8_ESM.pdf]

nature portfolio

Corresponding author(s): Aurel Holzschuh and Cristian Koepfli

Last updated by author(s): Jun 6, 2023

Reporting Summary

Nature Portfolio wishes to improve the reproducibility of the work that we publish. This form provides structure for consistency and transparency in reporting. For further information on Nature Portfolio policies, see our Editorial Policies and the Editorial Policy Checklist.

Statistics

For all statistical analyses, confirm that the following items are present in the figure legend, table legend, main text, or Methods section.

n/a	Confirmed	
☐	☒	The exact sample size (*n*) for each experimental group/condition, given as a discrete number and unit of measurement
☐	☒	A statement on whether measurements were taken from distinct samples or whether the same sample was measured repeatedly
☐	☒	The statistical test(s) used AND whether they are one- or two-sided<br>*Only common tests should be described solely by name; describe more complex techniques in the Methods section.*
☒	☐	A description of all covariates tested
☒	☐	A description of any assumptions or corrections, such as tests of normality and adjustment for multiple comparisons
☐	☒	A full description of the statistical parameters including central tendency (e.g. means) or other basic estimates (e.g. regression coefficient) AND variation (e.g. standard deviation) or associated estimates of uncertainty (e.g. confidence intervals)
☐	☒	For null hypothesis testing, the test statistic (e.g. *F*, *t*, *r*) with confidence intervals, effect sizes, degrees of freedom and *P* value noted<br>*Give P values as exact values whenever suitable.*
☐	☒	For Bayesian analysis, information on the choice of priors and Markov chain Monte Carlo settings
☒	☐	For hierarchical and complex designs, identification of the appropriate level for tests and full reporting of outcomes
☒	☐	Estimates of effect sizes (e.g. Cohen's *d*, Pearson's *r*), indicating how they were calculated

Our web collection on statistics for biologists contains articles on many of the points above.

Software and code

Policy information about availability of computer code

Data collection	Not applicable.
Data analysis	1. Haplotype calling and filtering was done using HaplotypR version 0.3 available at https://github.com/lerch-a/HaplotypR.git<br>2. Dcifer (version 1.2.0)<br>3. ade4 (version 1.7-20)<br>4. adegenet (version 2.1.8)<br>5. rmaverick (version 1.1.0)<br>6. Linkage ANalysis (LIAN version 3.7; available at http://guanine.evolbio.mpg.de/cgi-bin/lian/lian.cgi.pl/query)<br>7. igraph (version 1.3.5)<br>8. UpSetR (version 1.4.0)<br>9. ggplot2 (version 3.4.0)<br>10. fasterq-dump (v.2.10.8)<br>11. bwa (version 0.7.17-r1188)<br>12. samtools (version 1.10)<br>13. bcftools (version 1.10)<br>14. bedtools (version 2.30.0)<br>15. Swarm v2<br>16. vsearch (version 2.22.1)

For manuscripts utilizing custom algorithms or software that are central to the research but not yet described in published literature, software must be made available to editors and reviewers. We strongly encourage code deposition in a community repository (e.g. GitHub). See the Nature Portfolio guidelines for submitting code & software for further information.

Data

Policy information about availability of data

All manuscripts must include a data availability statement. This statement should provide the following information, where applicable:

- Accession codes, unique identifiers, or web links for publicly available datasets
- A description of any restrictions on data availability
- For clinical datasets or third party data, please ensure that the statement adheres to our policy

All raw sequencing data is available at the NCBI SRA (Accession number: PRJNA954767). De-identified datasets generated during the current study and used to make all figures are available as supplementary files or tables. This publication uses data from the MalariaGEN Plasmodium falciparum Community Project as described in 'Pf7: an open dataset of Plasmodium falciparum genome variation in 20,000 worldwide samples', MalariaGEN et al. Datasets extracted from PlasmoDB (https://plasmodb.org) can be accessed using the weblink. All analyses were completed in R version 4.1.2.

Research involving human participants, their data, or biological material

Policy information about studies with human participants or human data. See also policy information about sex, gender (identity/presentation), and sexual orientation and race, ethnicity and racism.

Reporting on sex and gender	This information has not been collected.
Reporting on race, ethnicity, or other socially relevant groupings	This information has not been collected.
Population characteristics	No human samples were collected as part of this study. This was a retrospective study of de-identified human samples. Samples were collected with informed consent from individuals as part of a previously conducted study. Only the parasite genetics were of interest. Other than sampling district, date of collection and travel history, human characteristics were not relevant to the data analysis. Therefore, this is technically not human subjects research but uses human material.
Recruitment	No recruitment of human subjects was performed as part of this study. This study involved evaluation of malaria parasite genomic data generated from DNA extracted from archived samples from completed studies
Ethics oversight	Ethical approval for this study was obtained from the Zanzibar Medical Research Ethics Committee (ZAMREC/0001/February/17), the Institutional Review Boards of Tulane University (Study Number: 993573) and of the Ifakara Health Institute (IHI/IRB/No: 003 – 2017), the Ethics Commission of North-western and Central Switzerland (EKNZ Reg-2017-00162), and the University of Notre Dame Institutional Review Board (approval no. 18-12-5029).

Note that full information on the approval of the study protocol must also be provided in the manuscript.

Field-specific reporting

Please select the one below that is the best fit for your research. If you are not sure, read the appropriate sections before making your selection.

☒ Life sciences ☐ Behavioural & social sciences ☐ Ecological, evolutionary & environmental sciences

For a reference copy of the document with all sections, see nature.com/documents/nr-reporting-summary-flat.pdf

Life sciences study design

All studies must disclose on these points even when the disclosure is negative.

Sample size	Sample size calculations were not performed, since this is a descriptive study. Rather, sample size was dictated by the number of cases with RDT+ malaria and the subsequently collected dried blood spots (DBS) from index cases and secondary cases within the two years collection period. The majority of all malaria-positive cases reported in the 5 districts included in the study were captured (including household members and neighboring households), and thus the sample size was deemed sufficient. All qPCR-positive DBS samples were attempted for sequencing. Final sample size was based on the number of parasite isolates with data in at least 10 loci and for which district-level geographic coordinates were available.
Data exclusions	Data was only excluded for not making it through the sequence filtering pipeline described in the paper. Nucleotide positions of the amplicons were only considered for haplotype calling, if they had a known SNP in PlasmoDB (https://plasmodb.org), or if they had a novel SNP that was dominant (>0.5 mismatch rate to the reference sequence) and was supported by at least two samples or replicates. Chimeric reads, singletons, and reads containing an insertion or deletion were excluded. Haplotype calling from the remaining reads required a within-host haplotype frequency of ≥1% and a minimum coverage of ≥10 reads per haplotype in ≥ 2 samples or replicates. Parasite isolates with data in less than 9 loci were excluded from all analyses.
Replication	The results have not been replicated in an independent dataset. However, samples were sequenced in duplicate in independent sequencing runs. A haplotype of a sample was only called if it was present in both replicates. True haplotypes should be detected in both replicates,

	unless the sequence depth is not sufficient for detecting a minority clone in one of the replicates (thus, haplotypes were only included if found in both replicates).
Randomization	This study was not a randomized controlled trial, and no randomization was used to group parasites. Parasite isolates were grouped spatially (i.e., by district, shehia, RACD cluster and household), temporally (i.e., date of collection) and travel history to determine genetic differentiation and relatedness/connectivity between and/or within parasite populations. For analyses of genetic differentiation (DAPC, STRUCTURE, Gst, Jost's D, and mLD), only monoclonal samples were included. For analysis of multidrug-resistant haplotypes, only monoclonal samples with complete allele calls for all seven drug resistance loci were included.
Blinding	This was a fixed sample and dataset, thus blinding was not necessary for this study. However, researchers carrying out the DNA extraction, PCR amplification, and sequencing were completely blinded to the sample identity.

Reporting for specific materials, systems and methods

We require information from authors about some types of materials, experimental systems and methods used in many studies. Here, indicate whether each material, system or method listed is relevant to your study. If you are not sure if a list item applies to your research, read the appropriate section before selecting a response.

Materials & experimental systems

n/a	Involved in the study
☒	☐ Antibodies
☒	☐ Eukaryotic cell lines
☒	☐ Palaeontology and archaeology
☒	☐ Animals and other organisms
☒	☐ Clinical data
☒	☐ Dual use research of concern
☒	☐ Plants

Methods

n/a	Involved in the study
☒	☐ ChIP-seq
☒	☐ Flow cytometry
☒	☐ MRI-based neuroimaging
